# A new high-throughput sequencing method for determining diversity and similarity of T cell receptor (TCR) α and β repertoires and identifying potential new invariant TCR α chains

**DOI:** 10.1186/s12865-016-0177-5

**Published:** 2016-10-11

**Authors:** Kazutaka Kitaura, Tadasu Shini, Takaji Matsutani, Ryuji Suzuki

**Affiliations:** 1 Repertoire Genesis Incorporation, 104 Saito-Bioincubator, 7-7-15, Saito-asagi, Ibaraki, Osaka 567-0085 Japan; 2 Department of Rheumatology and Clinical Immunology, Clinical Research Center for Rheumatology and Allergy, Sagamihara National Hospital, National Hospital Organization, Sagamihara, Japan; 3 BITS. Co., Ltd, Tokyo, Japan

**Keywords:** T cell receptor, Repertoire, Next generation sequencing, Immune profiling, Invariant TCRα

## Abstract

**Background:**

High-throughput sequencing of T cell receptor (TCR) genes is a powerful tool for analyses of antigen specificity, clonality and diversity of T lymphocytes. Here, we developed a new TCR repertoire analysis method using 454 DNA sequencing technology in combination with an adaptor-ligation mediated polymerase chain reaction (PCR). This method allows the amplification of all TCR genes without PCR bias. To compare gene usage, diversity and similarity of expressed TCR repertoires among individuals, we conducted next-generation sequencing (NGS) of TRA and TRB genes in peripheral blood mononuclear cells from 20 healthy human individuals.

**Results:**

From a total of 267,037 sequence reads from 20 individuals, 149,216 unique sequence reads were identified. Preferential usage of several V and J genes were observed while some recombinations of TRAV with TRAJ appeared to be restricted. The extent of TCR diversity was not significantly different between TRA and TRB, while TRA repertoires were more similar between individuals than TRB repertoires were. The interindividual similarity of TRA depended largely on the frequent presence of shared TCRs among two or more individuals. A publicly available TRA had a near-germline TCR with a shorter CDR3. Notably, shared TRA sequences, especially those shared among a large number of individuals’, often contained TCRα related with invariant TCRα derived from invariant natural killer T cells and mucosal-associated invariant T cells.

**Conclusion:**

These results suggest that retrieval of shared TCRs by NGS would be useful for the identification of potential new invariant TCRα chains. This NGS method will enable the comprehensive quantitative analysis of TCR repertoires at a clonal level.

**Electronic supplementary material:**

The online version of this article (doi:10.1186/s12865-016-0177-5) contains supplementary material, which is available to authorized users.

## Background

The term T cell repertoire describes a collection of lymphocytes characterized by T cell receptor (TCR) expression, which plays a critical role in antigen recognition. Since alterations of the T cell repertoire provide a significant indication of immune status in physiological and disease conditions, T cell repertoire analyses have been conducted for the identification of antigen-specific T cells involved in the development of disease and for the diagnosis of T lymphocyte abnormalities. Comparison of variable-region usage by fluorescence-activated cell sorter analysis using a large panel of antibodies specific for TCR variable regions [1–4], polymerase chain reaction (PCR) with multiple primers [5] or PCR-based enzyme-linked immunosorbent assay [6, 7] have been widely used to detect changes in T cell repertoire. Length distribution analysis known as CDR3 spectratyping is based on the addition of non-template nucleotides in V-(D)-J region and has been used to evaluate T cell clonality and diversity [8, 9]. To identify the antigen specificity of T cells further, PCR cloning of TCR clonotypes and subsequent sequence determination of the antigen recognition region, CDR3, have been required. These conventional approaches are commonly used but are time-consuming and a laborious way to study TCR repertoires.

In recent years, advances in high-throughput sequencing technologies known as next-generation sequencing (NGS) have rapidly progressed and enabled large-scale analysis of sequence data [10, 11]. Although several NGS-based TCR repertoire analysis systems have been developed by other researches, many amplification techniques are based on multiple PCR with different primers specific for each variable region. Thus, bias during PCR amplification is unavoidable since bias is most commonly due to differential hybridization kinetics among variable region-specific primers to different target genes. Correction and additional computational normalization methods are therefore required to minimize PCR bias when using multiple PCR assays [12]. The use of a single set of primers is a better way to achieve unbiased and quantitative amplification of all TCR genes including unknown variants where the 5’ ends of sequences are highly diverse. A single strand oligonucleotide anchor ligation to the 3’ end of cDNA with T4 RNA ligase [13], homopolymeric tailing of cDNA, 5’ rapid amplification of cDNA ends (RACE) [14] and template switching PCR (TS-PCR or SMART PCR) [15] have been used to analyze TCR repertoires [16, 17]. TS-PCR is simple and convenient but produces high levels of background amplification because TS primers non-specifically anneal to random regions in RNA or allow the repeated addition of TS primers [18, 19]. Thus, the current study describes an adaptor-ligation mediated PCR (AL-PCR) developed by the addition of an adaptor to the 5’ end of double stranded (ds) cDNA from TCR transcripts and subsequent PCR amplification with the adaptor primer and constant region-specific primer, as first reported by Tsuruta et al. [20, 21]. The adaptor ligation to blunt-ended ds cDNA is less influenced by the sequence of a particular cDNA while the efficiency of 5’ adaptor ligation with T4 RNA ligase is sequence dependent [22]. In addition, the ligation of dsDNA by T4 ligase is more efficient than ssDNA ligation with T4 RNA ligase in ligation anchored PCR (LA-PCR).

Various sequencing technologies such as Roche 454 (San Francisco, CA), Illumina (San Diego, CA), Ion-Torrent (Life Technologies, Grand Island, NY), SOLiD (Life Technologies), Helicos (Cambridge, MA) and PacBio (Menlo Park, CA) have been developed. Among these NGS platforms, the 454 DNA sequencing produces sequence reads ranging from 50 to 600 base pairs (bp) or more in length and sufficient read outputs, yet less reads per run than the Illumina. Long read sequencing allows determination of the full or near-complete length of TCR genes including V, D, J and C regions. Furthermore, recombinant TCR proteins can be easily produced by subsequent PCR cloning of the TCR genes. Therefore, we applied an adaptor-ligation mediated PCR method to NGS with 454 DNA sequencing.

Natural killer T (NKT) cells are a distinct T cell population with an important role in innate and adaptive immunity. NKT cells regulate a broad range of immune responses such as autoimmune diseases, tumor surveillance, and host defense against pathogenic infections. NKT cells express an invariant TCRα consisting of Vα24 and Jα18 that recognizes glycolipids presented by a non-classical major histocompatibility complex class I-related protein, CD1d [23]. Recently, mucosal-associated invariant T (MAIT) cells, which preferentially exist in mucosal tissues, were shown to be a unique T cell population expressing a semi-invariant TCRα consisting of Vα7.2 and Jα33. MAIT cells recognize microbial vitamin B metabolites presented by a non-classical MHC class I molecule, MHC-related protein 1 (MR1) [24]. These T cell populations bearing invariant TCRα play a pivotal role in immune regulation but it remains to be determined whether all invariant TCRα are expressed by these unique T cell populations.

In this study, we conducted NGS sequencing of TCR transcripts from 20 healthy individuals using a newly developed NGS-based TCR repertoire analysis. Initially, based on sequence read count, we examined usages of variable and joining regions, and further analyzed clonality and diversity in TCRα and β genes. Unique sequence reads identified using an originally developed gene analysis program were compared at a clonal level among healthy individuals. These results showed a similar usage of TRV and TRJ and similar extent of diversity of T cells among individuals. Interestingly, TCRβ reads were less shared among individuals while TCRα reads frequently contained shared sequences that overlapped between two or more individuals. Shared TCRα reads contained a high proportion of invariant TCRα indicating the presence of iNKT cells or MAIT cells.

In this report, we demonstrated that analysis of TCR genes shared among multiple individuals from NGS data provided significant information on invariant TCRs expressed by NKT cells and MAIT cells.

## Methods

### Isolation of peripheral blood mononuclear cells and RNA extraction

After obtaining written informed consent, whole blood was collected from 20 Japanese healthy individuals (age: 25–62 years old, median 31.5, male/female: 19/1, Additional file 1: Table S1). The study was approved by the ethics committees of the Clinical Research Center for Rheumatology and Allergy, Sagamihara National Hospital, National Hospital Organization. Ten ml of whole blood was collected into heparinized tubes. Peripheral blood mononuclear cells (PBMCs) were isolated with Ficoll-Paque PLUS™ (GE Healthcare Health Sciences, Uppsala, Sweden) density gradient centrifugation and washed with phosphate buffered saline (PBS). Cell numbers were counted and 1 × 10^6^ cells were used for RNA extraction. Total RNA was isolated and purified with RNeasy Lipid Tissue Mini Kit (Qiagen, Hilden, Germany) according to the manufacturer’s instructions. RNA amounts and purity were measured with Agilent 2100 bioanalyzer (Agilent Technologies, Palo Alto, CA).

### Unbiased amplification of TCR genes

One microgram of total RNA was converted to complementary DNA (cDNA) with Superscript III reverse transcriptase (Invitrogen, Carlsbad, CA). BSL-18E primer containing polyT_18_ and a *Not*I site was used for cDNA synthesis. After cDNA synthesis, double strand (ds)-cDNA was synthesized with *E. coli* DNA polymerase I (Invitrogen), *E. coli* DNA Ligase (Invitrogen), and RNase H (Invitrogen). ds-cDNAs were blunted with T4 DNA polymerase (Invitrogen). P10EA/P20EA adaptor was ligated to the 5’ end of the ds-cDNA and then cut with *Not*I restriction enzyme. After removal of adaptor and primer with MinElute Reaction Cleanup kit (Qiagen), PCR was performed using either TCR α-chain constant region-specific (CA1) or TCR β-chain constant region-specific primers (CB1) and P20EA (Table 1). PCR conditions were as follows: 95 °C (30 s), 55 °C (30 s), and 72 °C (1 min) for 20 cycles. The second PCR was performed with either CA2 or CB2 and P20EA primers using the same PCR conditions.

**Table 1:** Primers used in this study

Primer	Sequence	MID Tag
BSL-18E	AAAGCGGCCGCATGCTTTTTTTTTTTTTTTTTTVN	
P20EA	TAATACGACTCCGAATTCCC	
P10EA	GGGAATTCGG	
CA1	TGTTGAAGGCGTTTGCACATGCA	
CA2	GTGCATAGACCTCATGTCTAGCA	
CB1	GAACTGGACTTGACAGCGGAACT	
CB2	AGGCAGTATCTGGAGTCATTGAG	
HuVaF-01 ~ 10	**CCATCTCATCCCTGCGTGTCTCCGAC** TCAG-{MID}-ATAGGCAGACAGACTTGTCACTG	MID1 ~ MID11
HuVbF-01 ~ 10	**CCATCTCATCCCTGCGTGTCTCCGAC** TCAG-{MID}-ACACCAGTGTGGCCTTTTGGGTG	MID15 ~ MID24
B-P20EA	***CCTATCCCCTGTGTGCCTTGGCAGTC***TAATACGACTCCGAATTCCC	

V: A/C/G, N: A/C/G/T, Adaptor A and B sequences were typed in bold and bold italic, respectively. A key sequence (TCAG) was underlined. The following MID Tag sequences were used for identification of sample source. MID1: ACGAGTGCGT, MID2: ACGCTCGACA, MID3: AGACGCACTC, MID4: AGCACTGTAG, MID5: ATCAGACACG, MID6: ATATCGCGAG, MID7: CGTGTCTCTA, MID8: CTCGCGTGTC, MID10: TCTCTATGCG, MID11: TGATACGTCT, MID15: TACGACGTA, MID16: TCACGTACTA, MID17: CGTCTAGTAC, MID18: TCTACGTAGC, MID19: TGTACTACTC, MID20: ACGACTACAG, MID21: CGTAGACTAG, MID22: TACGAGTATG, MID23: TACTCTCGTG, MID24: TAGAGACGAG

### Amplicon sequencing by Roche 454 sequencing system

Amplicons for NGS were prepared by amplification of the second PCR products using P20EA primer and fusion Tag primer (Table 1). The fusion Tag primers consisting of an A-adaptor sequence (CCATCTCATCCCTGCGTGTCTCCGAC), 4 base sequence key (TCAG), multiple identifier (MID) Tag sequence (10 nucleotides), and TCR constant region-specific sequence were designed according to the manufacturer’s instructions. After PCR amplification, amplicons were separated and evaluated by agarose gel electrophoresis. The resulting fragment (~600 bp) was removed from the gel and purified with QIAEX II gel extraction kit (Qiagen). The amount of purified amplicon was quantified by Quant-iT™ PicoGreen® dsDNA Assay Kit (Life Technologies, Carlsbad, CA). Each amplicon obtained with a different fusion Tag primer from 10 healthy individuals was mixed at equal molar concentrations. Emulsion PCR (emPCR) was performed using the amplicon mixtures with GS Junior Titanium emPCR Lib-L kit (Roche 454 Life Sciences, Branford, CT) according to the manufacturer’s instructions.

### Assignment of TRV and TRJ segments

All sequence reads were classified by MID Tag sequences. Artificially added sequences (Tag, adaptor, and key) and sequences with low quality scores were removed from both terminals of sequence reads using software installed on the 454 sequencing system. The remaining sequences were used for assignment of TRAV and TRAJ for TCR α sequences, and TRBV and TRBJ for TCR β sequences. Assignment of sequences was performed by determining sequences with the highest identity in a data set of reference sequences for 54 TRAV, 61 TRAJ, 65 TRBV and 14 TRBJ genes including pseudogenes and open reading frame (ORF) reference sequences available from the international ImMunoGeneTics information system® (IMGT) database (http://www.imgt.org). Data processing, assignment, and data aggregation were automatically performed using repertoire analysis software originally developed by our group (Repertoire Genesis, RG). RG implemented a program for sequence homology searches using BLATN, an automatic aggregation program, a graphics program for TRV and TRJ usage, and CDR3 length distribution. Sequence identities at the nucleotide level between query and entry sequences were automatically calculated. Parameters that increased sensitivity and accuracy (E-value threshold, minimum kernel, high-scoring segment pair (HSP) score) were carefully optimized for respective repertoire analysis.

### Data analyses

Nucleotide sequences of CDR3 regions ranged from conserved Cysteine at position 104 (Cys104) of IMGT nomenclature to conserved Phenylalanine at position 118 (Phe118) and the following Glycine (Gly119) was translated to deduced amino acid sequences. A unique sequence read (USR) was defined as a sequence read having no identity in TRV, TRJ and deduced amino acid sequence of CDR3 with the other sequence reads. The copy number of identical USR were automatically counted by RG software in each sample and then ranked in order of the copy number. Percentage occurrence frequencies of sequence reads with TRAV, TRAJ, TRBV and TRBJ genes in total sequence reads were calculated.

### Retrieval of shared USRs among samples

To retrieve shared sequences among samples, a concatenate string of “TRV gene name”_” deduced amino acid sequence of CDR3 region”_” TRJ gene name” of individual USR (for example: TRBV1_CASTRVVJFG_TRBJ2-5) was used as a TCR identifier. The TCR identifier in a sample was retrieved in read data sets from all the other samples.

### Diversity indices and Similarity index

To estimate TCR diversity in deep sequence data, several diversity indices, Simpson’s index and Shannon-Weaver index were calculated using a function “diversity” of the vegan package in the R program. These indices were calculated based on the number of species per sample and the number of individuals per sample as measures for biological diversity in ecology. In deep sequence data, USR and copy number were used for species and individuals, respectively. Simpson’s index (1-λ) was defined as:

$$ 1-\lambda =1-{\displaystyle \sum_{i=1}^S\left(\frac{n_i\left({n}_i-1\right)}{N\left(N-1\right)}\right)}, $$

where N is the total number of sequence reads, n_*i*_ is the copy number of *i*th USR, and *S* is the species number of USR. This value ranges from 0 to 1, where the maximum number 1 means high levels of diversity and 0 indicates low diversity. Simpson’s reciprocal index (1/λ) was also calculated as the inverse of λ. The Shannon-Weaver index (*H*’) was used for the diversity index and was defined as:

$$ H\mathit{\hbox{'}}=-{\displaystyle \sum_{i-1}^S\frac{n_i}{N}} \ln \frac{n_i}{N} $$

where N is the total number of sequence reads, n_*i*_ is the number of *i*th USR, and *S* is the species number of USR. These diversity indices should be biased by differences in read numbers among samples. Therefore, the number of sequence reads was standardized for each sample down to the smallest number of sequence reads [25]. To standardize sample size, repeated random resampling 1000 times without replacement and calculation of the diversity index were performed using the R program. The median of their indices was used to determine the diversity index for the sample.

To estimate the similarity of TCR repertoires between healthy individuals, the Morisita-Horn index was calculated using the function “vegdist” in the vegan package of the R program. The Morisita-Horn index (*C*_*H*_) was defined as:

$$ {C}_H=\frac{2{\displaystyle {\sum}_{i=1}^S{x}_i{y}_i}}{\left(\frac{{\displaystyle {\sum}_{i=1}^S{x}_i^2}}{X^2}+\frac{{\displaystyle {\sum}_{i=1}^S{y}_i^2}}{Y^2}\right)XY} $$

where x_*i*_ is the number of *i*th USR in the total X reads of one sample, y_*i*_ is the number of *i*th USR in the total Y reads of another sample, and *S* is the number of USR. To standardize the sample size, repeated random resampling 1000 times without replacement and calculation of similarity index were performed using the R program [26]. Median values were used for similarity indexes between a pair of samples.

### Statistics

Statistical significances were tested by the nonparametric Mann–Whitney *U-*test using GraphPad Prism software (version 4.0, San Diego, CA). A value of *P* < 0.05 was considered statistically significant.

## Results

### Repertoire analysis software

The cloud-based software platform developed in this study, RG, is a high-speed, accurate and convenient computing system for TCR repertoire analysis. RG provides an integrated software package for 1) assignment of V, D and J segments, 2) calculation of sequence identity, 3) extraction of CDR3 sequences, 4) counting identical reads, 5) amino acid translation, 6) frame analysis (stop and frame-shift), and 7) CDR3 length analysis. After uploading sequencing data from the NGS sequencer, V, D and J segments can be identified based on their sequence similarity with optimized parameters. Read counts are automatically summarized and then processing data, summary tables, and graphs can be easily downloaded.

### Read number, error rate, and unproductive reads

We performed high-throughput sequencing of TRA and TRB genes in PBMCs from 20 healthy individuals. A total of 172,109 and 91,234 sequence reads were assigned for TRA and TRB repertoire analyses, respectively using the RG program (Additional file 1: Tables S2 and S3). A total of 94,928 and 57,982 unique sequence reads (UDR) were identified in TRA and TRB, respectively. The number of nucleotide sequences per read obtained by Roche 454 sequencing were ~400 bp in length (mean bp length ± SD, TRA: 407.4 ± 35.4, TRB: 409.4 ± 37.8), indicating these sequences were long enough to identify TCR genes ranged from V region to J regions. To evaluate the accuracy and quality of NGS sequencing, we calculated the frequency of mismatched nucleotides between query and reference sequences as the error rate. Error rates were 0.72 ± 0.18 % for TRAV, 0.54 ± 0.08 % for TRAJ, 0.70 ± 0.15 % for TRBV and 0.50 ± 0.12 % for TRBJ (Additional file 1: Table S4). These error rates were slightly lower than in a previous study reporting a mean error rate of 1.07 % for 454-sequences [27]. The error rates were significantly higher in V regions than in J regions (AV vs. AJ: *P* < 0.05, BV vs. BJ: *P* < 0.0001), indicating higher sequence reliability in regions close to sequencing primers. Occurrence frequency of unproductive reads carrying a stop codon or a shift of reading frame in CDR3 regions (out-of-frame) was calculated (Additional file 1: Table S5). There was no significant difference in the percentage of occurrence frequency of unproductive unique sequence reads between TRA and TRB (31.2 ± 7.0 % vs. 29.3 ± 7.9 %, *P* = 0.31). Regarding the error rates, frequencies of mismatched nucleotides were significantly higher in out-of-frame reads than in in-frame reads in all regions of TRAV, TRAJ. TRBV and TRBJ (Additional file 1: Table S4). The differences were quite significant in J regions (in-frame vs. out-of-frame, TRAJ: 0.37 vs. 1.08 %, TRBJ: 0.33 vs. 1.16 %). This suggested that sequencing errors occurred in J regions adjacent to CDR3 cause frequently frame-shift of coding sequence.

### Expression of TCR genes including pseudogenes and ORF

**Fig. 1:**
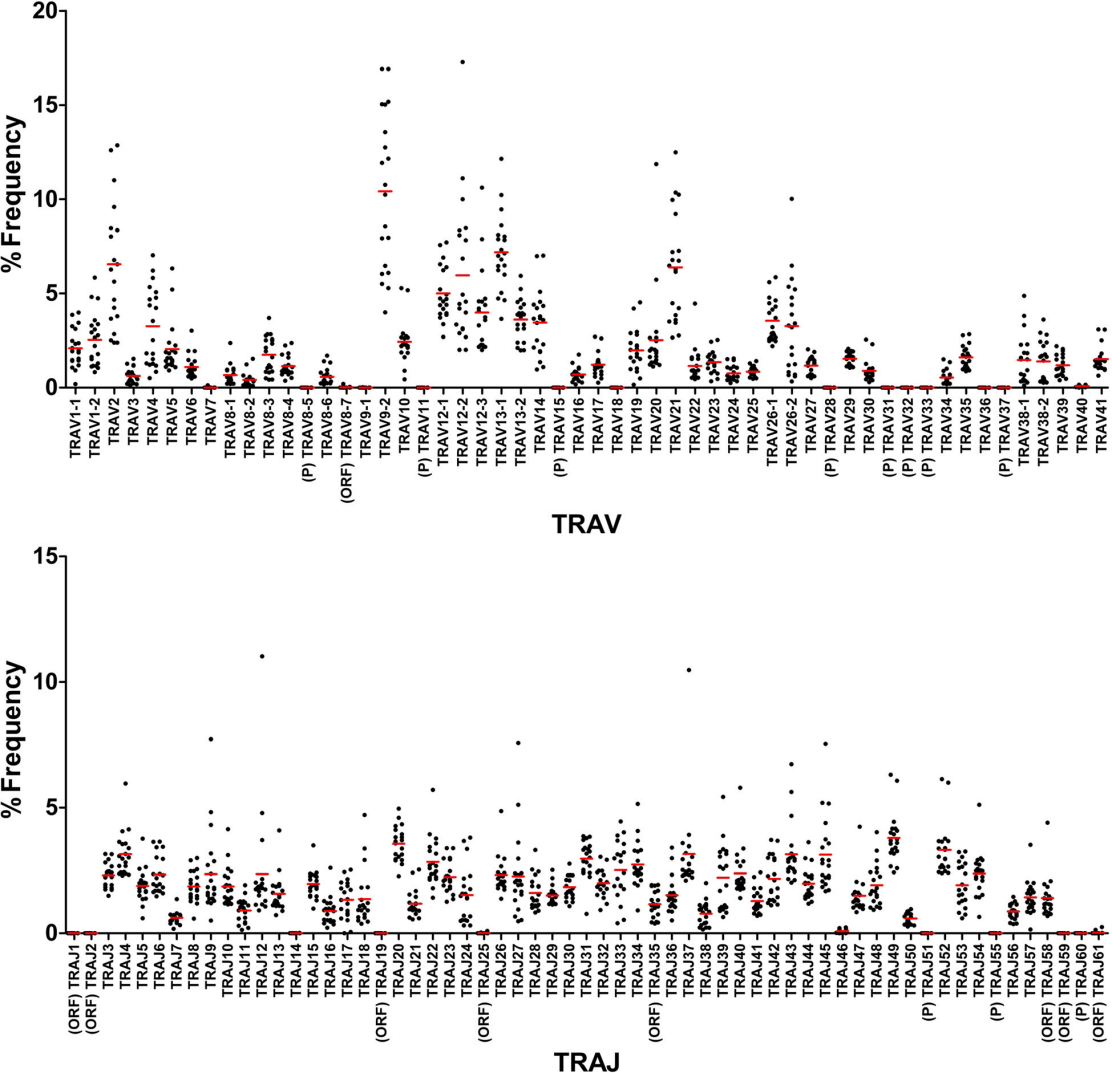
Usage of TRAV and TRAJ in 20 healthy individuals. The numbers of TCR sequences bearing respective TRAV and TRAJ were counted. The percentage frequencies of 54 TRAV and 61 TRAJ were calculated and are shown as a scatter plot. Each dot indicates the percentage frequency of TRAV or TRAJ in each individual. Bars in red indicate the mean values of 20 individuals. (P): pseudogene, (ORF): open reading frame

**Fig. 2:**
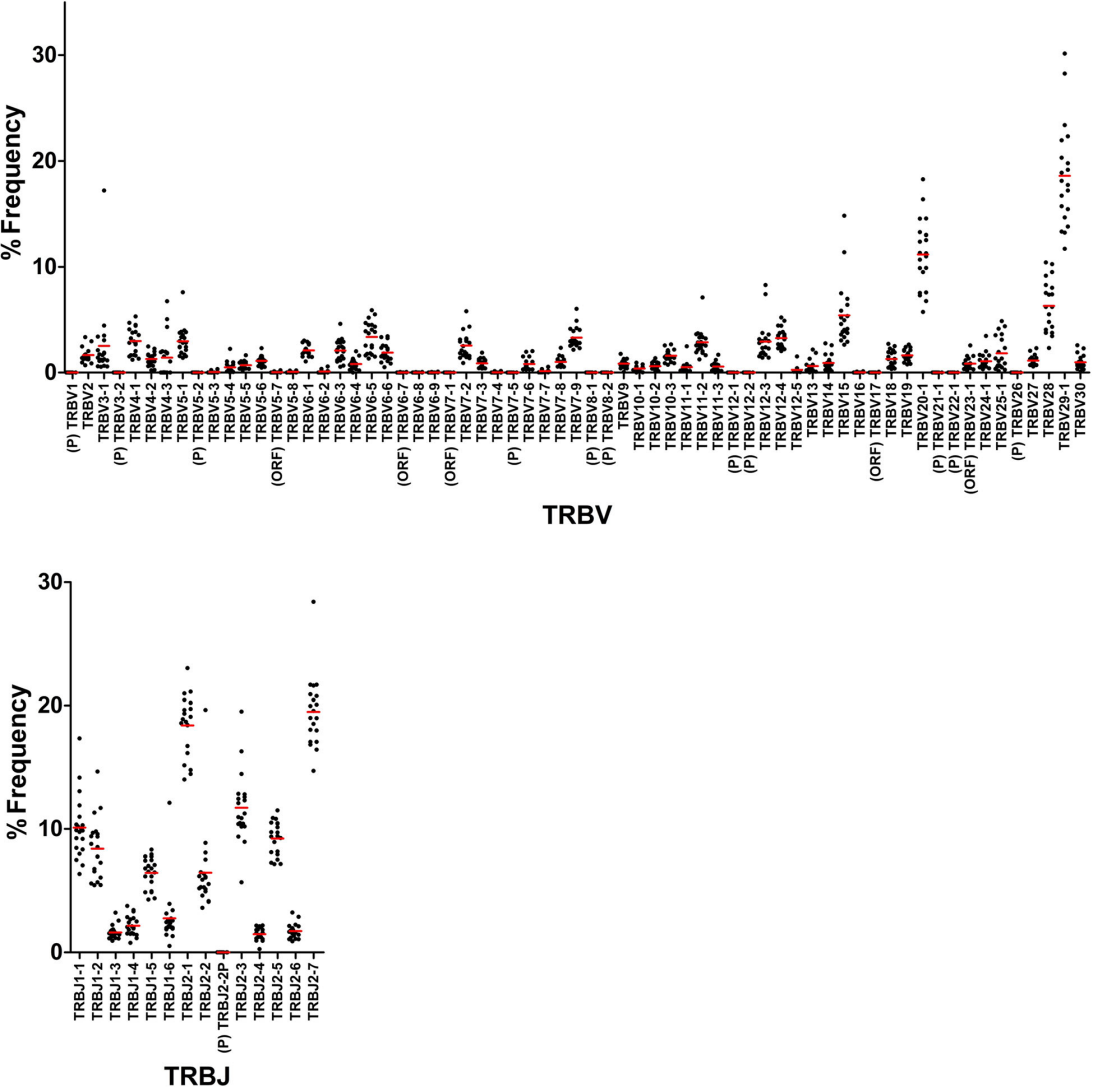
Usage of TRBV and TRBJ in 20 healthy individuals. The percentage frequencies of 65 TRBV and 14 TRBJ are shown as a scatter plot. Each dot indicates the percentage frequency of TRBV or TRBJ in each individual. Bars in red indicate the mean values. (P): pseudogene, (ORF): open reading frame

To determine usages of TRV and TRJ genes in TCR sequence reads, copy number (read number) of USRs bearing respective TRV or TRJ were counted. Individual USRs were ranked by order of copy number and the percentage frequency of respective TRV and TRJ were calculated (Figs. 1 and 2). Regarding TRA repertoires, eight pseudogenes (AV8-5, AV11, AV15, AV28, AV31, AV32, AV33 and AV37) were not expressed in healthy individuals. AV8-7, classified as an ORF (defined based on alterations in splicing sites, recombination signals and/or regulatory elements by IMGT), were slightly expressed (43 reads in 11 of 20 individuals). Expression of AV18 and AV36 (classified as functional genes) was not observed in healthy individuals. In addition, the functional genes, AV7 and AV9-1, were poorly expressed in 1 individual (9 reads) and 2 individuals (3 reads), respectively. Of eight AJ genes (AJ1, AJ2, AJ19, AJ25, AJ35, AJ58, AJ59 and AJ61) classified as ORF genes, the expression of AJ35 and AJ58 were observed in all 20 individuals. Of these, AJ25 and AJ61 were slightly expressed in 3 individuals (21 reads) and 7 individuals (35 reads), respectively. AJ1, AJ2, AJ19 and AJ59 were not present in any individuals. There was no expression of the three pseudogenes, AJ51, AJ55 and AJ60, in any individuals. The functional gene AJ14 was detected in only 3 reads from 3 individuals.

For TRB genes, there was no expression of 11 pseudogenes (BV1, BV3-2, BV5-2, BV7-5, BV8-1, BV8-2, BV12-1, BV12-2, BV21-1, BV22-1 and BV26) in healthy individuals. Of five ORF genes, BV5-7 (32 reads in 13 individuals), BV6-7 (13 reads in 8 individuals) and BV17 (3 reads in 1 individual) were poorly expressed. The BV7-1 ORF gene was not observed in any individuals while BV23-1 was expressed in all 20 individuals. Regarding BJ genes, there was no expression of the BJ2-2P pseudogene.

### Infrequent recombination of TRAV and TRAJ

**Fig. 3:**
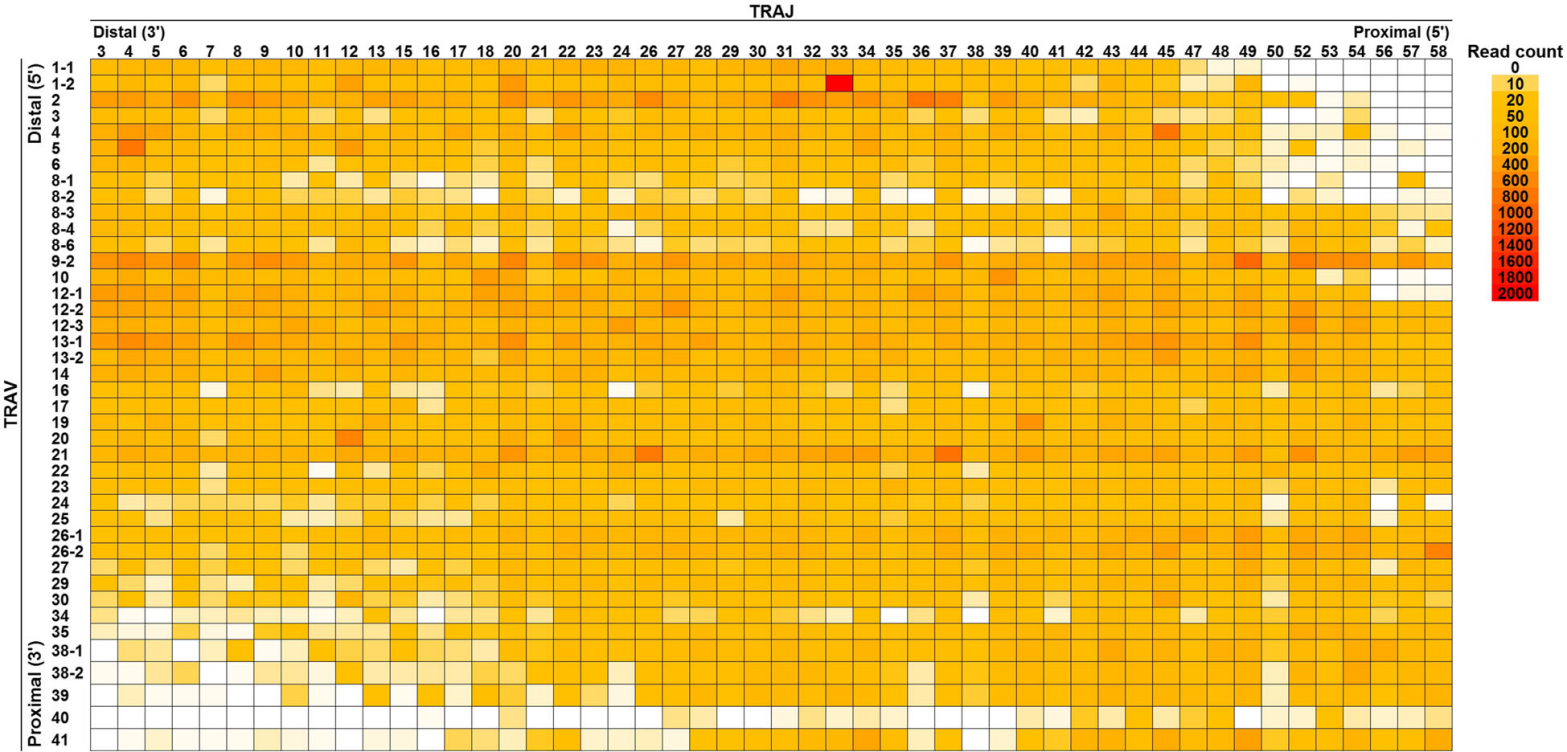
Heatmap representation of gene recombination of TRAV with TRAJ in pooled read data from 20 healthy individuals. The numbers of TCR sequence reads bearing respective gene recombination of TRAV and TRAJ were counted. The occurrence tendency of recombination is visualized by heat map presentation of the number of each recombination. Color in each pixel indicates the number of each recombination. For TRAV, 8 pseudogenes (TRAV8-5, TRAV11, TRAV15, TRAV28, TRAV31, TRAV32, TRAV33, and TRAV37), 1 ORF (TRAV8-7), and poorly expressed genes (TRAV7, TRAV9-1, TRAV18 and TRAV36) were excluded. For TRAJ, 3 pseudogenes (TRAJ51, TRAJ55, and TRAJ60), 6 ORFs (TRAJ1, TRAJ2, TRAJ19, TRAJ25, TRAJ59, and TRAJ61), and poorly expressed genes (TRAJ14 and TRAJ46) were excluded. Two ORFs (TRAJ35 and TRAJ48) found to be expressed were included. A heat map representation of the 2050 recombination events (41 TRAV × 50 TRAJ) is shown

Gene recombination of 41 TRAV with 50 TRAJ (except for pseudogenes, ORF, and poorly expressed genes) were capable of generating a total of 2050 AV-AJ recombinations (Fig. 3), of which 1969 AV-AJ recombinations (96.0 %) were detected in 20 individuals. This indicated that almost all AV-AJ recombinations were used in TCR transcripts without restriction. Notably, AV1-1–AV6 genes were recombined less preferentially with AJ50–AJ58 genes and similarly, recombination of AV35–AV41 genes with AJ3–AJ16 was rarely observed. Given the chromosomal location of these gene segments, these results showed that AV-AJ recombinations occurred infrequently between proximal AV genes and distal AJ genes and between distal AV genes and proximal AJ genes. For TRB, 650 gene recombinations were generated with 50 BV (except for 11 pseudogenes and 5 ORFs) and 13 BJ genes (except for a pseudogene), of which 605 BV-BJ (93.1 %) were used in 20 individuals. There was no restriction for combinations of TRBV with TRBJ.

### Preferential usage of TRV and TRJ repertoires in healthy individuals

To reveal TRV and TRJ usage in whole TCR transcripts, the occurrence frequency of USRs bearing respective TRV or TRJ were calculated (Figs. 1 and 2). Preferential usage in several TRAV genes was observed in TRAV2 (11S1, nomenclature by Arden et al. [28]), TRAV9-2 (AV21S1), TRAV13-1 (AV8S1) and TRAV21 (AV23S1). These preferential usages were similar to a previous result obtained using a hybridization-based quantitation assay [6]. Several TRBV genes were abundantly used in the TRBV repertoire. The top three TRBV29-1 (BV4S1 by Arden), TRBV20-1 (BV2S1) and TRBV28 (BV3S1) accounted for approximately one third of the total sequence reads. This was similar to results from our previous study using the microplate hybridization assay (MHA) [6]. Gene usage varied considerably among the TRBJ genes. TRBJ2-1 and TRBJ 2–7 were highly expressed while the expressions of TRBJ1-3, TRBJ1-4, TRBJ1-6, TRBJ2-4 and TRBJ2-6 were low. Next, to examine if the preferential usage of TRV and TRJ are due to peripheral selection, we compared usages of TCR between in-frame and out-of-frame reads (Additional file 1: Figure S3). As a whole, we observed little difference in the usages of TRAV, TRAJ, TRBV and TRBJ between in-frame and out-of-frame reads. On the other hand, particularly TRAV26-2, TRAJ4, TRAJ37, TRBV23-1, TRBJ1-4 and TRBJ2-2 were more frequently used in out-of-frame reads than in-frame reads.

### Three-dimensional (3D) view of TCR repertoire usage

**Fig. 4:**
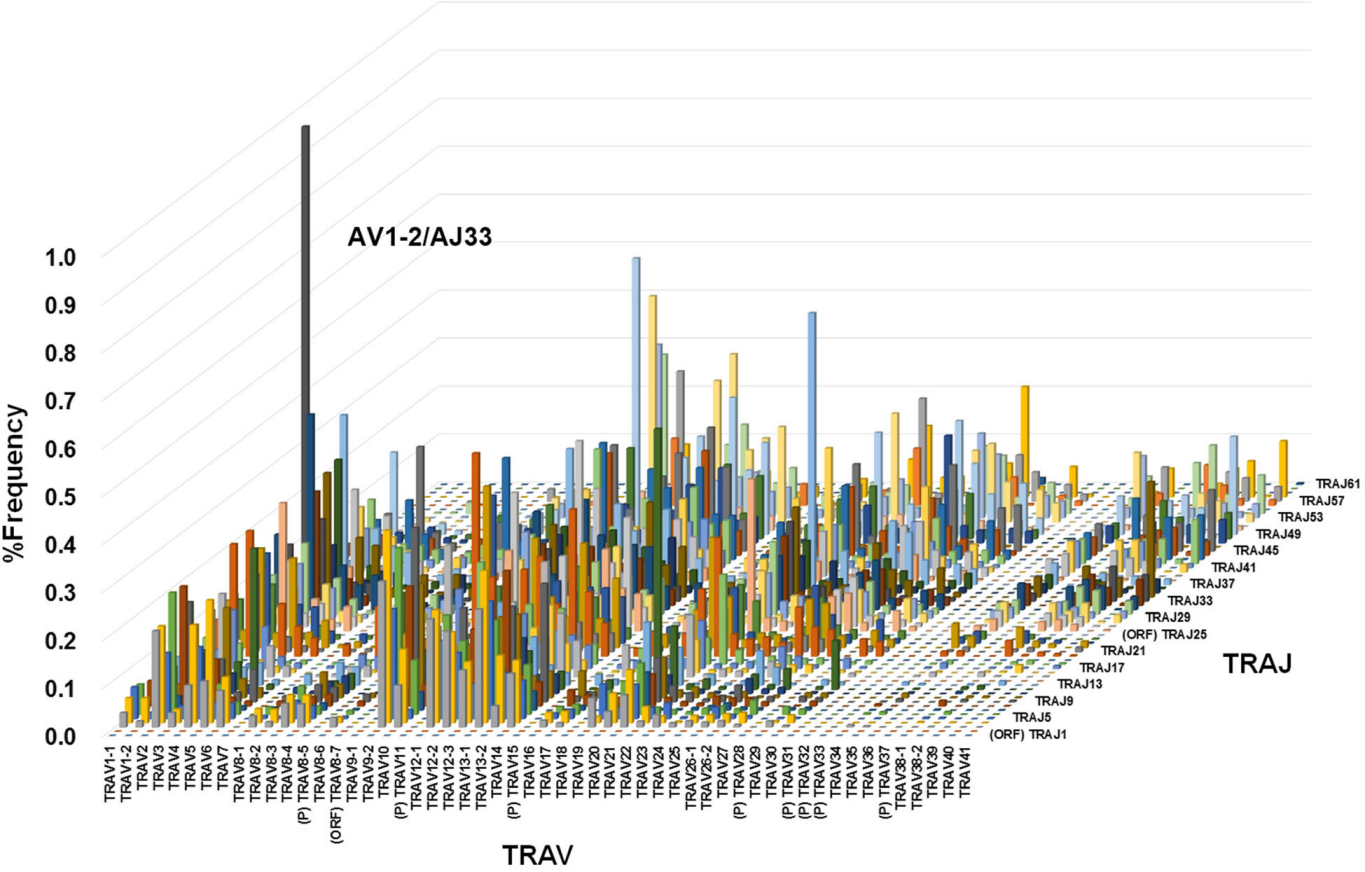
3D image of TRA repertoires. The numbers of TCR sequence reads bearing a given gene recombination of TRAV with TRAJ were counted. The mean percentage frequencies of 3294 (54 TRAV × 61 TRAJ) in 20 healthy individuals are shown as a 3D bar graph. X-axis and Y-axis indicate TRAV and TRAJ, respectively. Recombination of TRAV1-2 with TRAJ33 (AV1-2/AJ33) was the most expressed (0.99 ± 0.85). (P): pseudogene, (ORF): open reading frame

**Fig. 5:**
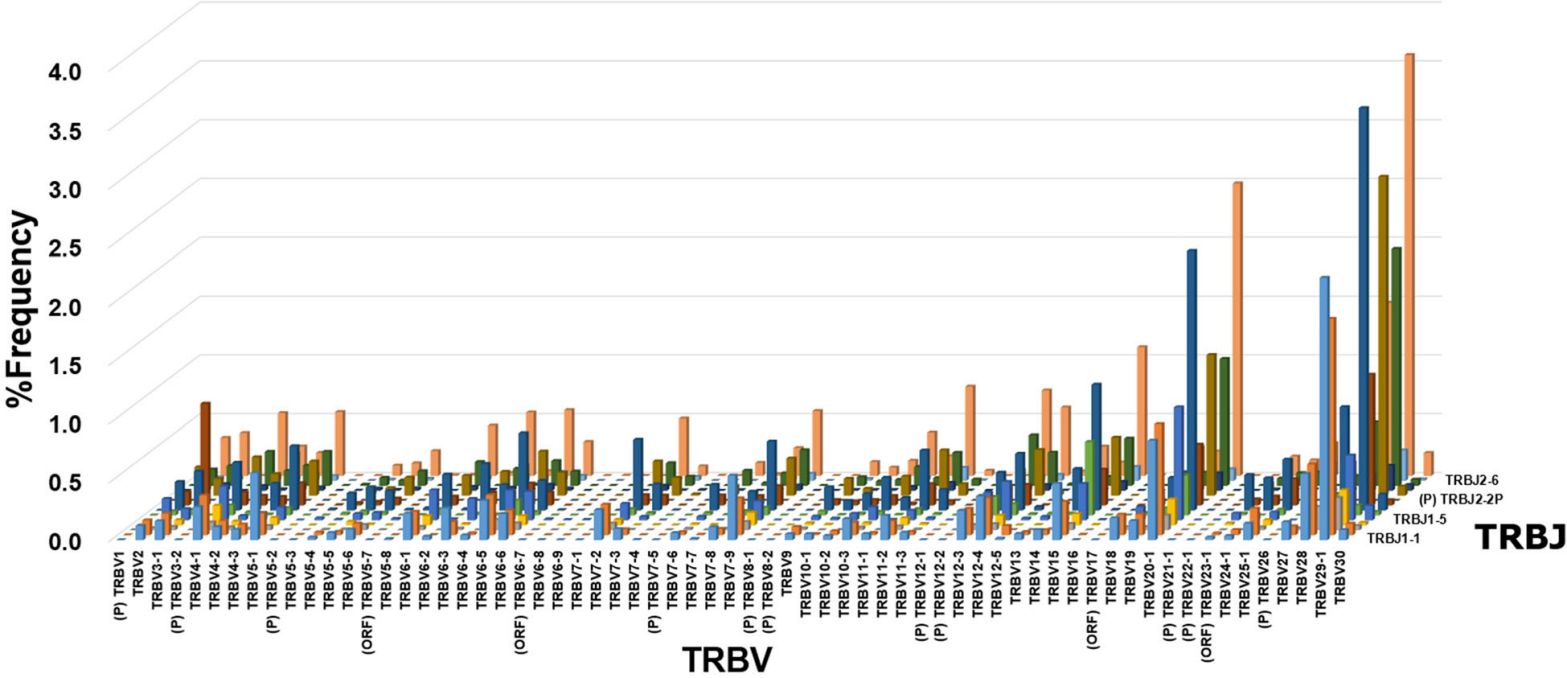
3D image of TRB repertoires. The numbers of TCR sequence reads bearing a given gene recombination of TRBV with TRBJ were counted. The mean percentage frequencies of 910 (65 TRBV × 14 TRBJ) in 20 healthy individuals are shown as a 3D bar graph. X-axis and Y-axis indicate TRBV and TRBJ, respectively. (P): pseudogene, (ORF): open reading frame

To visualize the usage of TCRs bearing a combination of TRV with TRJ genes, we produced 3D pictures of the TCR repertoires (Figs. 4 and 5). The advantage of 3D images is that the dominance of certain combination of TRV with TRJ genes as well as the extent of diversity of TCR can easily be observed. For TRB, there was little preferential usage of recombination between TRBJ and TRBV genes. The frequency of each recombination depended on the usage of TRBV or TRBJ. BV29-1/BJ2-7, BV29-1/BJ2-1, BV29-1/BJ2-3 and BV20-1/BJ2-7 were frequently used in all combinations while others were expressed at a low frequency. In contrast, 3D imaging of the TRA repertoire indicated a low level of expression with a wide distribution of TRAV and TRAJ. The occupancy was lower than 1 % for all combinations. Of note, TCR reads bearing AV1-2 and AJ33 were highly expressed in all healthy individuals (mean ± SD: 0.99 ± 0.85).

### Digital CDR3 length distribution

**Fig. 6:**
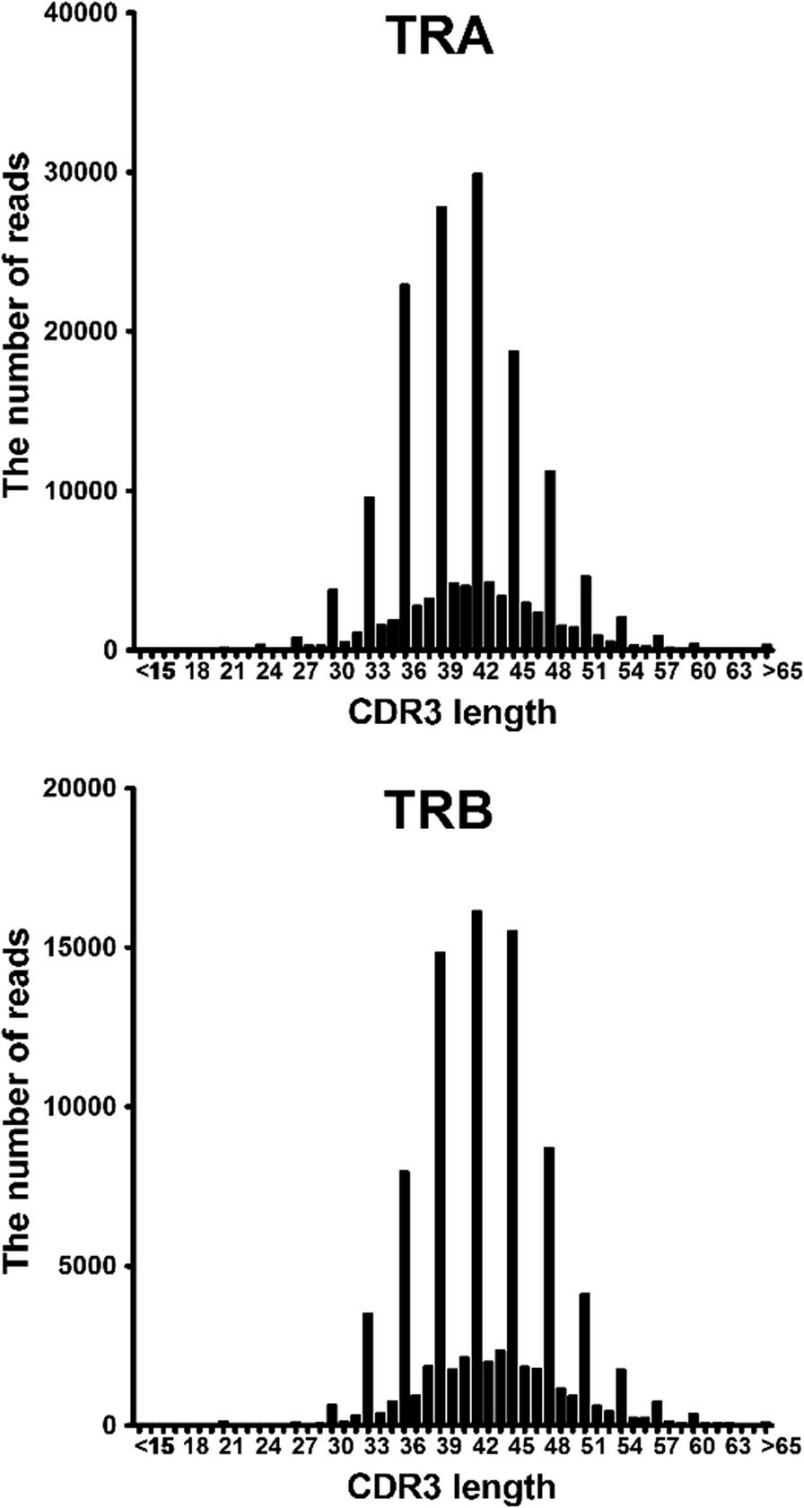
Digital CDR3 length distribution of TRA and TRB. Lengths of CDR3 were determined in 172,109 TRA and 94,928 TRB sequence reads obtained from the pooled data of 20 individuals. Length of nucleotide sequences from conserved cysteine at position 104 (Cys104) of IMGT nomenclature to conserved phenylalanine at position 118 (Phe118) were automatically calculated using RG software. Distribution of CDR3 length in TRA (upper) and TRB (lower) is shown as a histogram

The analysis of CDR3 length distribution, termed CDR3 size spectratyping [29, 30] or immunoscope analysis [31, 32], has been effectively used to estimate the diversity of the TCR repertoire. This technique is based on actual peak distributions of PCR amplicons including CDR3 sequences by gel electrophoresis. In the current study, the length of the determined nucleotide sequences of TCRs ranging from conserved Cys104 (IMGT nomenclature) to conserved phenylalanine at position 118 (Phe118) were calculated automatically. This provided a visually easy way to estimate the diversity and clonality of TCRs by using NGS data. RG can produce figures depicting the digital CDR3 length distribution for each V region. CDR3 length distribution of both TRA and TRB were similar to a normal distribution but were not completely symmetrical (Fig. 6). CDR3 length was shorter in TRA than in TRB (mean ± SD: 41.2 ± 8.3 vs. 42.8 ± 6.1) and TRA had more positive skewness than TRB (skewness index: 11.1 vs. 5.41), indicating the distribution in TRA was concentrated to the left. In addition, TRA had more positive kurtosis than in TRB, showing a high degree of peakedness in TRA (kurtosis index: 282.4 vs. 176.7).

### Diversity of TRA and TRB repertoires

**Fig. 7:**
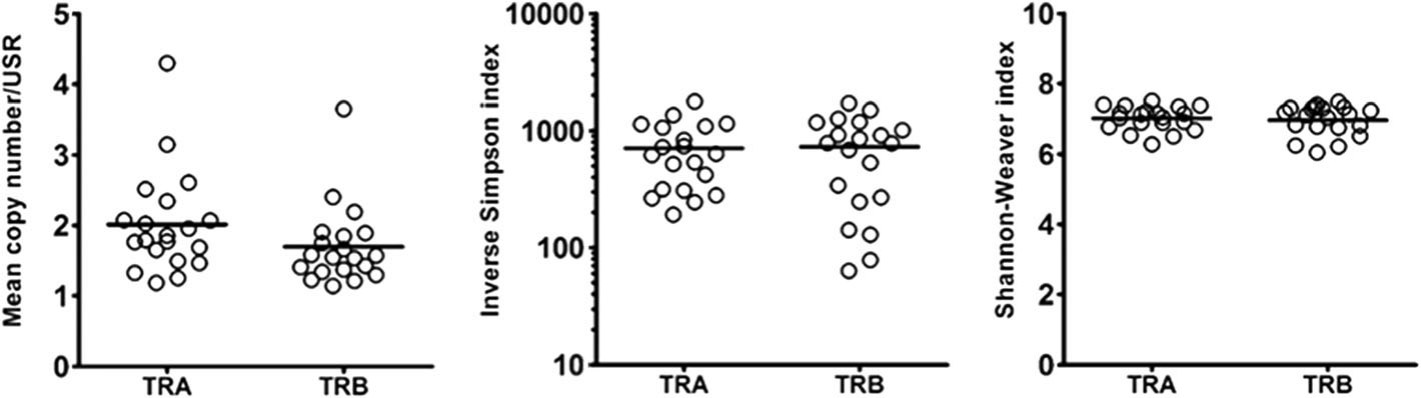
Diversity of TRA and TRB repertoires in healthy individuals. Copy number (read number) of unique sequence reads (USR) was calculated. Mean copy number per unique sequence read in each individual are shown as open circle (*left*). Inverse Simpson index (*middle*) and Shannon-Weaver index (*right*) were calculated with R program according to formulas described in Materials and Methods. Each open circle indicates the index of an individual. There were no significant differences in mean copy number, inverse Simpson index and Shannon-Weaver index between TRA and TRB

To demonstrate diversity of the TCR repertoire, we calculated the mean copy number of USR and diversity indices such as the Simpson index and Shannon-Weaver index (Fig. 7). The mean copy number of USR was not significantly different between TRA and TRB (2.0 ± 0.72 vs. 1.70 ± 0.57). In addition, there was no significant difference in Simpson inverse index (D) and Shannon-Weaver index (H) between TRA and TRB (D: 710.3 ± 433.0 vs. 729.7 ± 493.9, H: 7.02 ± 0.33 vs. 6.97 ± 0.43). These results indicated that immune diversity for TCRα and β in healthy individuals was not different. Next, we examined whether the diversity was different between in-frame (productive) read and out-of-frame (unproductive) reads (Additional file 1: Figure S4). The result indicated that Shannon diversity indices were significantly higher in in-frame reads than in out-of-frame reads (TRA: 7.37 ± 0.72 vs. 6.81 ± 0.49, *p* < 0.05, TRB: 7.05 ± 0.64 vs. 6.46 ± 0.60, *P* < 0.005). However, there was no difference in the inverse Simpson index between in-frame and out-of-frame reads. Furthermore, we examined if T cell diversity was correlated with age (Additional file 1: Figure S5). There was a significant correlation of Shannon index in TRA with age with a Spearman’s correlation of −0.46 (*P* < 0.05) but no significant correlation of the other indices with age. Overall, these results showed tendencies for inverse relationship of TCR diversity with age.

### Similarity of TRA and TRB repertoire among healthy individuals

To reveal correlations of gene usage between individuals, percentage frequencies of each TRV and TRJ were plotted between all pairs of individuals by scatterplot (Additional file 1: Figure S1). Spearman’s correlation coefficient between each pair was calculated. The concordance correlation coefficients were lower in TRAV than in TRBV (mean ± SD, 0.86 ± 0.059 in TRAV, 0.89 ± 0.038 in TRBV, *P* < 0.001) and the values were lower in TRAJ than in TRBJ (0.74 ± 0.095 in TRAJ, 0.91 ± 0.063 in TRBJ, *P* < 0.001) (Additional file 1: Figure S2). These results indicated that the expression levels of TRV and TRJ between healthy individuals were more similar among individuals in TRB compared with TRA.

**Fig. 8:**
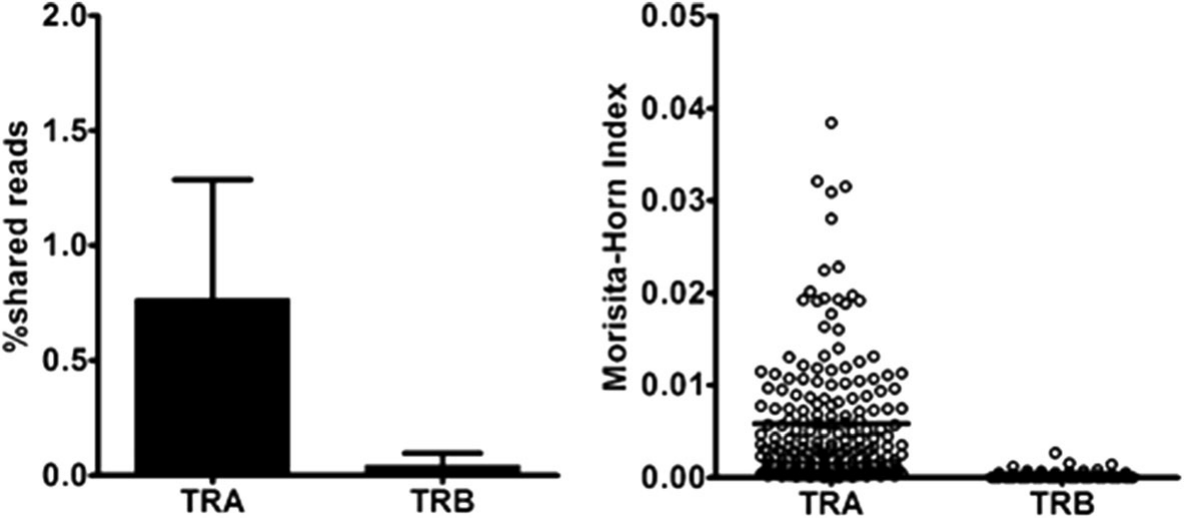
Similarity of TRA and TRB repertoires in healthy individuals. The occurrence frequency of TCR sequence reads shared between all pairs of 20 individuals was calculated (Additional file 1: Tables S5 and S6). Mean percentage frequency of shared reads were compared between TRA and TRB (left, *n* = 380). Similarity index, Morisita-Horn index, was calculated with the R program according to a formula described in Materials and Methods. There were significant differences in the frequency of shared reads and similarity index between TRA and TRB (*P* < 0.001 and *P* < 0.001, respectively, Mann–Whitney *U-*test)

To evaluate the potential similarity of TCR repertoires at the clonal level between healthy individuals, we retrieved TCR sequence reads that were shared among individuals. The number of shared TCR reads between all pairs of individuals was counted and their occurrence frequencies were calculated (Additional file 1: Tables S6 and S7). The mean frequency was significantly higher in TRA compared with TRB (0.76 ± 0.52 vs. 0.040 ± 0.057, *n* = 380, *P* < 0.001) (Fig. 8), indicating the TRA repertoire contains more common TCR reads among individuals than TRB does. A similarity index, Morisita-Horn index, was significantly larger for TRA than TRB (0.0058 ± 0.0069 vs. 0.000096 ± 0.00029, *n* = 190, *P* < 0.001). These results clearly indicated that TRA repertoires were more similar between healthy individuals compared with TRB repertoires.

### Shared TCR sequences among healthy individuals

**Fig. 9:**
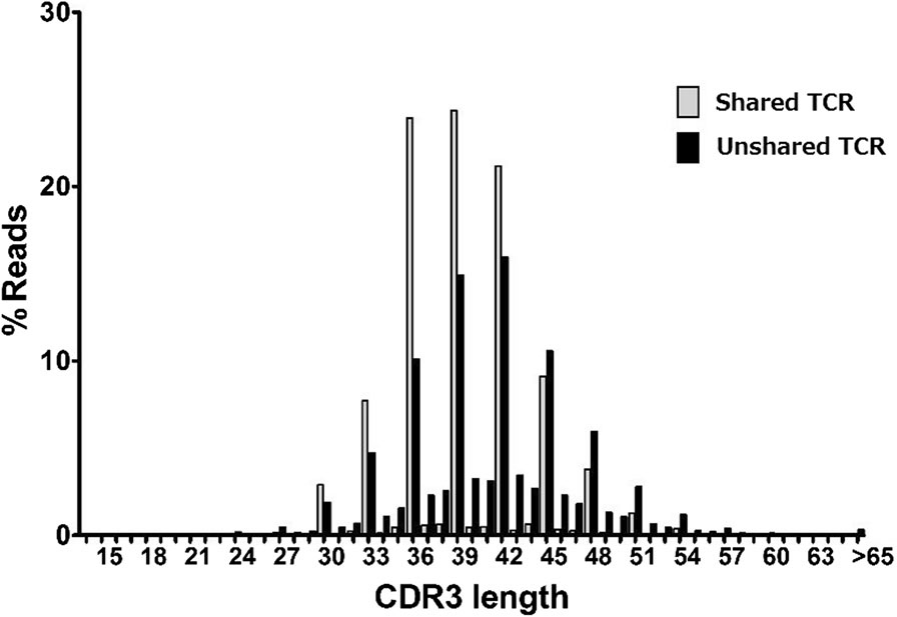
Shared TCR had shorter CDR3 lengths than private TCR. CDR3 lengths were calculated with 7237 USRs of Shared TCR (Grey) and 83,997 USRs of Unshared TCR (Black). Percentage frequencies of USRs in each CDR3 length are plotted as a bar graph. Median values of CDR3 length in Shared and unshared TCR are 39 and 42, respectively

A small number of TCR sequences were shared between different healthy individuals. In contrast, most TCR sequences were specific for each healthy individual. To identify shared TCR sequences in 20 healthy individuals, we retrieved TRA and TRB reads shared between two or more healthy individuals. In 20 individuals, 3041 shared TRA and 206 shared TRB sequences were obtained from 90,643 and 57,982 USRs, respectively (Table 2). Shared TRA were more frequent in PBLs from healthy individuals than TRB were. Shared TRB sequences were obtained from two to four individuals while the shared TRA sequences were observed in 16 individuals. These results indicated that shared TRA sequences were more commonly used by individuals but that the TRB repertoire was more specific for each individual. Furthermore, the occurrence frequencies per individual of TCR sequences shared between a pair of individuals were significantly higher for TRA (7.9 %) than TRB (0.7 %). To characterize the shared TRA sequences, we compared the CDR3 length between shared and unshared TRA sequences and observed that shared TRA had a shorter CDR3 length than unshared TRA (median: 39 vs. 42) (Fig. 9).

**Table 2:** Numbers of TRA and TRB sequences shared among multiple healthy individuals

No. of individuals	No. of shared TCRs	
	TRA	TRB
2	2390	196
3	424	9
4	125	1
5	47	0
6	23	0
7	9	0
8	4	0
9	5	0
10	5	0
11	2	0
12	0	0
13	3	0
14	1	0
15	1	0
16	2	0
17	0	0
18	0	0
19	0	0
20	0	0
Total	3041	206

Numbers of identical TCR sequences observed in multiple healthy individuals (2–20 individuals) were counted

### TCRs shared among multiple individuals frequently contain invariant TCRα chains

Shared TRA were frequently observed in PBLs from healthy individuals. To determine the origin of the shared TRA, we investigated CDR3 sequences of shared TRA reported previously. Interestingly, shared TRA sequences that were common to multiple individuals contained a high proportion of TCRα related with invariant TCRα indicative of iNKT cells or MAIT cells (Table 3). It was reported that MAIT cells express TRAV1-2 and TRAJ33 while iNKT express TRAV10 and TRAJ18. Many shared TRAs used TRAV1-2 and TRAJ33 with different CDR3 sequences. The total percentage frequencies of MAIT TRAs bearing TRAV1-2 and TRAJ33 and iNKT TRAs bearing TRAV10 and TRAJ18 were 0.82 ± 0.72 % and 0.15 ± 0.41 % per individual, respectively. Of 55 shared TRA sequences, 22 (40 %) MAIT and one (1.8 %) iNKT sequences were observed in six or more individuals (Table 3). The rate increased with the number of overlapping individuals. Germline-like CDR3 sequences that had no amino acid sequences altered from germline sequences were observed in 27 (71 %) of 38 shared TRAs except for MAIT (TRAV1-2-TRAJ33) and NKT (TRAV10-TRAJ18).

**Table 3:** Invariant TCRs observed in shared TRA sequences

Shared Individuals^a^	TRAV	TRAJ	CDR3^b^	Invariant TCR^c^
16	TRAV1-2	TRAJ33	CAVRDSNYQLIW	MAIT-like
16	TRAV1-2	TRAJ33	CAVMDSNYQLIW	MAIT-like
15	TRAV1-2	TRAJ33	CAVLDSNYQLIW	MAIT-like
14	TRAV1-2	TRAJ12	CAVMDSSYKLIF	MAIT-like
13	TRAV1-2	TRAJ33	CAVTDSNYQLIW	MAIT-like
13	TRAV1-2	TRAJ20	CAVRDGDYKLSF	MAIT-like
13	TRAV1-2	TRAJ33	CAVKDSNYQLIW	MAIT-like
11	TRAV1-2	TRAJ33	CAAMDSNYQLIW	MAIT-like
11	TRAV1-2	TRAJ33	CAALDSNYQLIW	MAIT-like
10	TRAV9-2	TRAJ20	CALNDYKLSF	
10	TRAV1-2	TRAJ33	CAVVDSNYQLIW	MAIT-like
10	TRAV10	TRAJ18	CVVSDRGSTLGRLYF	iNKT-like
10	TRAV1-2	TRAJ33	CAVIDSNYQLIW	MAIT-like
10	TRAV13-2	TRAJ9	CAENTGGFKTIF	
9	TRAV1-2	TRAJ33	CAVSDSNYQLIW	MAIT-like
9	TRAV9-2	TRAJ53	CALSGGSNYKLTF	
9	TRAV2	TRAJ36	CAVEDQTGANNLFF	
9	TRAV9-2	TRAJ45	CALSDSGGGADGLTF	
9	TRAV1-2	TRAJ20	CAVRDRDYKLSF	MAIT-like
8	TRAV1-2	TRAJ33	CAGMDSNYQLIW	MAIT-like
8	TRAV21	TRAJ20	CAVNDYKLSF	
8	TRAV1-2	TRAJ33	CAPMDSNYQLIW	MAIT-like
8	TRAV1-2	TRAJ33	CASMDSNYQLIW	MAIT-like
7	TRAV12-2	TRAJ30	CAVNRDDKIIF	
7	TRAV13-2	TRAJ53	CAENSGGSNYKLTF	
7	TRAV1-2	TRAJ33	CAPLDSNYQLIW	MAIT-like
7	TRAV9-2	TRAJ53	CALNSGGSNYKLTF	
7	TRAV12-1	TRAJ20	CVVNDYKLSF	
7	TRAV9-2	TRAJ20	CALSSNDYKLSF	
7	TRAV13-1	TRAJ15	CAASNQAGTALIF	
7	TRAV12-1	TRAJ49	CVVNTGNQFYF	
7	TRAV12-1	TRAJ27	CVVNTNAGKSTF	
6	TRAV2	TRAJ9	CAVEDTGGFKTIF	
6	TRAV1-2	TRAJ33	CAVEDSNYQLIW	MAIT-like
6	TRAV21	TRAJ26	CAVDNYGQNFVF	
6	TRAV9-2	TRAJ53	CALSDSGGSNYKLTF	
6	TRAV21	TRAJ12	CAVMDSSYKLIF	
6	TRAV2	TRAJ9	CAVNTGGFKTIF	
6	TRAV1-2	TRAJ33	CAVRDGNYQLIW	MAIT-like
6	TRAV9-2	TRAJ8	CALNTGFQKLVF	
6	TRAV13-2	TRAJ44	CAENTGTASKLTF	
6	TRAV1-2	TRAJ33	CAATDSNYQLIW	MAIT-like
6	TRAV12-2	TRAJ15	CAVNQAGTALIF	
6	TRAV13-2	TRAJ42	CAENYGGSQGNLIF	
6	TRAV21	TRAJ30	CAVLNRDDKIIF	
6	TRAV2	TRAJ26	CAVEDNYGQNFVF	
6	TRAV12-2	TRAJ20	CAVNDYKLSF	
6	TRAV12-1	TRAJ31	CVVNNARLMF	
6	TRAV2	TRAJ26	CAVDNYGQNFVF	
6	TRAV2	TRAJ3	CAVDSSASKIIF	
6	TRAV9-2	TRAJ23	CALIYNQGGKLIF	
6	TRAV9-2	TRAJ9	CALNTGGFKTIF	
6	TRAV13-2	TRAJ39	CAENNAGNMLTF	
6	TRAV1-2	TRAJ12	CAVLDSSYKLIF	MAIT-like
6	TRAV1-2	TRAJ12	CAAMDSSYKLIF	MAIT-like

^a^Number of individuals in which respective shared TRAs were detected; ^b^Non-germline amino acid sequences generated by nucleotide addition/deletion are underlined; ^c^MAIT-like: mucosal-associated invariant T cells-like (TRAV1-2 and TRAJ33, TRAV1-2 and TRAJ12 and TRAJ33 and TRAJ20, iNKT-like: invariant natural killer T cells (TRAV10 and TRAJ18)

## Discussion

High-throughput sequencing technologies have taken a great leap forward with the development of a wide variety of NGS platforms. NGS facilitates the acquisition of an enormous amount of sequence data but still requires PCR amplification or gene enrichment to sequence genes of specific interest instead of the entire genome or gene library. For heterogeneous TCR or BCR genes generated by rearrangement of many gene segments, multiplex PCR with many sets of gene-specific primers have been widely used. However, the use of multiple primers causes amplification bias between respective genes, hampering the accurate estimation of gene frequency. Here, we used an unbiased PCR technique, an adaptor-ligation mediated PCR, for NGS-based TCR repertoire analysis. The method uses a single set of primers to avoid PCR bias by competition between primers. Therefore, it is better suited to estimate accurately the abundances of respective TCR genes from a wide variety of samples.

We comprehensively examined TRA and TRB repertoires at the clonal level from a large number of individuals (*n* = 20) and evaluated a large number amount of sequence data (total 149,216 unique sequence reads from 267,037 sequence reads). Thus, this study precisely revealed the normal range of gene usage as well the extent of diversity and similarity of TCR repertoires in healthy individuals. Compared with the Illumina NGS platform [16, 17, 33], sample sequence reads were less numerous but were longer and of higher quality. Using the Illumina platform, a different sequence depth among CDR3 contig generated from many shotgun reads may make it difficult to determine the frequency of TCR clonotypes. However, all TCR sequences were determined from a single read and had long sequences that covered the entire region of CDR3, V and J (Mean ~400 bp, Additional file 1: Tables S1 and S2). Direct analysis from read sequences without assembly is likely to reflect accurately the actual frequencies of TCR clonotypes. Error rates in TCR sequences were slightly less than a previous report showing a mean error rate for 454-sequences of 1.07 % [27], suggesting high levels of accuracy and quality irrespective of nested PCR. Homopolymeric stretches within coding regions occur typically with the 454-sequence methodology, causing a frame shift in coding sequence. This leads to higher rate of production of out-of-frame reads in the 454-sequence compared with other sequence platforms. Bolotin et al. has previously reported that higher percentage of mismatch-containing sequencing in the illumina than in the Roche 454 and Ion Torrent datasets (3.2, 1.4 and 1.2 %) [34]. The error rate obtained in this study seems to be relatively lower than that in the previous report, even though our data showed higher error rate in out-of-frame than in-frame reads. This supports that the error rates obtained are lower than in the illumina although our results did not provide a direct evidence. Furthermore, the assignment and aggregation software, RG, can rapidly summarize usage as well as recombination usage of TRV and TRJ. This integrated analysis easily allows the detection of preferential usage of a given TRV and/or TRJ and therefore it will be useful for studying immune responses by antigen-specific T cells.

Unlike widely used multiplex PCR methods that typically require compensation for PCR bias [12], the AL-PCR method is supposed to accurately estimate TCR repertoires without the compensation. High expression levels of TRBV18 (BV18S1, Arden’s nomenclature), TRBV19 (BV17S1) and TRBV7-9 (BV6S5) as well as low expression levels of TRBV20-1 (BV2S1), TRBV28 (BV3S1) and TRBV29-1 (BV4S1) were reported in CD4+ and CD8+ cells by multiplex PCR [35]. However, flow cytometry analysis showed that TRBV20 and TRBV29 were abundantly expressed in PBLs [1, 36, 37]. To examine difference of accuracy between AL-PCR and multiplex PCR in detail, we compared usage of TRBV obtained with either AL-PCR or multiplex PCR with FACS data reported previously by van den Beemd et al. [1]. The result indicated that AL-PCR method was better correlated with FACS method than Multiplex PCR method, suggesting that the AL-PCR method with a set of universe primers enables us to accurately analyze TCR repertoires. In addition, our results of TCR repertoires are similar to a previous report [38]. Therefore, this method will provide direct, accurate and dependable results of TCR repertoires.

By comparison to large number of healthy individuals, it has been disclosed that disease patients with X-linked agammaglobulinemia [39] or Common Variable Immune Deficiency (CVID) [40] had skewed and contracted TCR repertoires. It is important to clarify TCR repertoires of healthy individuals in considering how much disease patients differ from normal. Regarding usages of TRV and TRJ repertoires, we observed preferential usages of TRV and TRJ in peripheral bloods from healthy individuals. Similar usages between in-frame (productive) and out-of-frame (unproductive) reads suggests that the preferential usages are unlikely due to peripheral selection. Given the preferential usage was observed in immature T cells [41], this is likely to be influenced by genetic factors such as recombination process.

Recombination usage exhibited infrequent recombinations of AJ-proximal 3’ AV segment to AV-distal 3’ AJ segment and AJ-distal 5’ AV segment to AV-proximal 5’ AJ segments. In gene rearrangement of the TCRαδ locus, activation of the TCRα enhancer (Eα) and the T early activation (TEA) promoter initiate primary rearrangement of proximal TRAV and TRAJ segments. Subsequent secondary rearrangement occurs using 5’ distal TRAV and distal 3’ TRAJ genes [42–45], resulting in the restricted usage of TRA repertoires (model of sequential bidirectional recombination) [46]. However, all TRAV genes can recombine with TRAJ genes in secondary rearrangement by the model of locus contraction and DNA looping formation [47]. Although there was inefficient recombination of distal-proximal and proximal-distal TRAV-TRAJ genes, TRAJ usage was not limited over all TRAV but rather was equally distributed. This suggests that the frequency of recombination varies dependent on the location of TRAV and probably depends on the ability of loop formation between TRAV and TRAJ loci.

Potential TCR diversity generated by recombination and nucleotide addition/deletion has been estimated to be up to 10^15^ [48]. By NGS-based estimation, TRB diversity was estimated to be 3–4 × 10^6^ [33] or approximately 1 × 10^6^ in humans [17]. Furthermore, diversity of TRA is 50 % of that of TRB in humans [49]. In mice, TRA diversity was suggested to be 0.79 × 10^4^ [44] or 1.18 × 10^4^ [50] and is 10-fold lower than TRB diversity. This lower diversity of TRA might be caused by a difference in recombination processes between TRA and TRB. However, our results showed a similar extent of diversity between TRA and TRB as evaluated by Simpson and Shannon-Weaver indices. Similarly, Wang et al. reported that TCR diversity was estimated to be equal between TRA and TRB (0.47 × 10^6^ vs. 0.35 × 10^6^) [51, 52]. Contrary to previous reports obtained using limited number of sequences, large-scale sequencing suggests that the repertoire size for TRA generated by V-J recombination is comparable with that for TRB by V-D-J recombination.

As for TCR diversity, productive TCR had more diverse than unproductive one. Only a portion of T cells produce both productive and unproductive TCRs. This difference might be depend on the number of reads obtained from the library. Also, there was a correlation between the diversity and age. This is consistence with the previous report that age-related decrease in TCR repertoire was found [53]. Diverse T cells are generated from thymus and the thymic involution occurs with age. The decrease in TCR diversity in periphery is likely due to the age-dependent decrease in thymic T cell regeneration.

Of note, we found that TRA repertoires were considerably similar between individuals. This was mainly due to the presence of shared TCR sequences between two or more individuals. It has been reported that shared TCRβ amino acid sequences have fewer additions in their nucleotide sequences [54, 55]. Random nucleotide addition and deletion mediated by terminal deoxynucleotidyl transferase occurs during TCR rearrangement, resulting in a remarkable increase in diversity of the CDR3 region. However, the shared TCRs appeared to have germline-like CDR3 sequences that did not undergo such modifications (Table 3). Furthermore, the shared TCRs contained many TCR clonotypes with a shorter CDR3 length. These results suggest that the frequent occurrence of shared TRAs is likely to be caused by a difference in the inherent recombination mechanism from TRB (V-J vs. V-D-J).

It is noteworthy that the shared TRA were present in a large number of individuals. We unexpectedly found that the shared TRA contained a high rate of TCRα related with invariant TCRα derived from MAIT cells or iNKT cells. These functionally important T cells have homogenous TCRα and diverse TCRβ. MAIT cells express a canonical TCRα including TRAV1-2 (Vα7.2)-TRAJ33 (Jα33) and are preferentially localized in the gut lamina propria [56, 57] and a TCRα bearing TRAV1-2-TRAJ12 and TRAV1-2-TRAJ20 [58, 59]. MAIT cells recognize vitamin B2 metabolites presented by MR1, non-classical MHC class I molecule [24, 57]. Furthermore, CD1d-restricted iNKT cells express an invariant TRAV10 (Vα24)-TRAJ18 (Jα18) chain and semi-invariant TRBV25-1 (Vβ11) [60] and recognize glycolipids such as α-galactosyl ceramide, self-glycolipid, or isoglobotrihexosyl ceramide [61]. Both cell types play an essential role in the regulation of immune responses against infections, tumors, autoimmune diseases, and tolerance induction [23]. Frequencies of MAIT and iNKT cells obtained in this study were consistent with previous reports showing MAIT cells expanded up to 1–4 % of peripheral blood T cells [62] and that iNKT cells accounted for 0.2 % of total PBMCs [63]. Interestingly, different types of shared sequences bearing TRAV1-2 (for example, TRAV1-2-TRAJ12, TRAV1-2-TRAJ20) and several shared TRA sequences other than the well-known MAIT and iNKT sequences exist. Therefore, NGS-based repertoire analysis is useful for both estimating the frequency of MAIT or iNKT cells as well as identifying potential new invariant TCRα chains. Further identification and verification is required to identify potential novel invariant TCRα.

## Conclusion

We developed a new NGS-based TCR repertoire analysis method and thereby clearly revealed comparable diversity and different interindividual similarity between TRA and TRB. Shared TRA sequences contained frequent functionally significant T cell subpopulations, MAIT and iNKT cells. The approach to seeking shared TCR by NGS would be useful for identification of potential new invariant TCRα chains. This useful technology for TCR repertoire analysis will enable us to reveal antigen-specific T cells relevant to the pathogenesis of human disease and contribute to studies of innate and adaptive immunity.

## Additional file

Additional file 1:
**Table S1.** Age, gender and chronic illness of 20 healthy individuals. **Table S2.** Numbers of unique reads, reads and nucleotides in TRA reads obtained from PBMCs of 20 healthy individuals. **Table S3.** Numbers of unique reads, reads and nucleotides in TRB reads obtained from PBMCs of 20 healthy individuals. **Table S4.** Percentage of mismatched nucleotides in in-frame and out-of-frame TCR sequences. **Table S5.** Occurrence frequency of out-of-frame unique sequence reads in TRA and TRB. **Table S6.** Percentage frequency of shared TRA reads between all pairs of individuals. **Table S7.** Percentage frequency of shared TRB reads between all pairs of individuals. **Figure S1.** Correlation of gene usage of TRAV, TRAJ, TRBV and TRBJ between healthy individuals. **Figure S2.** Concordance correlation coefficient in TRAV, TRAJ, TRBV and TRBJ. **Figure S3.** Comparison of TCR usages between in-frame and out-of-frame reads sequences. **Figure S4.** Diversity indices of in-frame and out-of-frame TRA and TRB. **Figure S5.** Correlation of TCR diversity with age. **Figure S6.** Correlation of TCR usage from a published FACS data with AL-PCR and Multiplex PCR. (DOCX 548 kb)

## Abbreviations

CDR: Complementarity determining region
TRA: T cell receptor alpha
TRAJ: T cell receptor alpha joining
TRAV: T cell receptor alpha variable
TRB: T cell receptor beta
TRBJ: T cell receptor alpha joining
TRBV: T cell receptor beta variable
