# Supplementary material for: A new high-throughput sequencing method for determining diversity and similarity of T cell receptor (TCR) α and β repertoires and identifying potential new invariant TCR α chains
Source: BMC Immunol. 2016 Oct 11;17:38. doi: 10.1186/s12865-016-0177-5 (PMC5059964; doi:10.1186/s12865-016-0177-5)
Supplement: Additional file 1: — Table S1. Age, gender and chronic illness of 20 healthy individuals. Table S2. Numbers of unique reads, reads and nucleotides in TRA reads obtained from PBMCs of 20 healthy individuals. Table S3. Numbers of unique reads, reads and nucleotides in TRB reads obtained from PBMCs of 20 healthy individuals. Table S4. Percentage of mismatched nucleotides in in-frame and out-of-frame TCR sequences. Table S5. Occurrence frequency of out-of-frame unique sequence reads in TRA and TRB. Table S6. Percentage frequency of shared TRA reads between all pairs of individuals. Table S7. Percentage frequency of shared TRB reads between all pairs of individuals. Figure S1. Correlation of gene usage of TRAV, TRAJ, TRBV and TRBJ between healthy individuals. Figure S2. Concordance correlation coefficient in TRAV, TRAJ, TRBV and TRBJ. Figure S3. Comparison of TCR usages between in-frame and out-of-frame reads sequences. Figure S4. Diversity indices of in-frame and out-of-frame TRA and TRB. Figure S5. Correlation of TCR diversity with age. Figure S6. Correlation of TCR usage from a published FACS data with AL-PCR and Multiplex PCR. (DOCX 548 kb) [file 12865_2016_177_MOESM1_ESM.docx]

| **Table S1. Age, gender and chronic illness of 20 healthy individuals** | | | |
| --- | --- | --- | --- |
| **Healthy individual** | **Age** | **Gender** | **Chronic illness** |
| H001 | 43 | male | no |
| H002 | 62 | male | no |
| H003 | 32 | male | no |
| H004 | 29 | male | no |
| H005 | 30 | male | no |
| H006 | 29 | male | no |
| H007 | 31 | male | no |
| H008 | 58 | male | no |
| H009 | 35 | male | no |
| H010 | 28 | male | no |
| H011 | 28 | female | no |
| H012 | 39 | male | no |
| H013 | 42 | male | no |
| H014 | 48 | male | no |
| H015 | 32 | male | no |
| H016 | 25 | male | no |
| H017 | 46 | male | no |
| H018 | 25 | male | no |
| H019 | 28 | male | no |
| H020 | 26 | male | no |
| Median | 31.5 |  |  |

| **Table S2. Numbers of unique reads, reads and nucleotides in TRA reads obtained from PBMCs of 20 healthy individuals** | | | | |
| --- | --- | --- | --- | --- |
| **Healthy individual** | **Total number of unique reads** | **Total number of reads** | **Total number of nucleotides** | **Mean number of nucleotides per read** |
| H001 | 5,902 | 8,805 | 3,732,329 | 423.9 |
| H002 | 2,809 | 5,812 | 2,477,523 | 426.3 |
| H003 | 1,707 | 7,334 | 3,269,817 | 445.8 |
| H004 | 5,586 | 6,981 | 3,047,583 | 436.6 |
| H005 | 3,250 | 5,815 | 2,507,968 | 431.3 |
| H006 | 4,267 | 7,043 | 3,052,709 | 433.4 |
| H007 | 5,467 | 6,462 | 2,784,462 | 430.9 |
| H008 | 3,350 | 6,206 | 2,700,726 | 435.2 |
| H009 | 4,966 | 7,267 | 3,119,902 | 429.3 |
| H010 | 5,019 | 6,641 | 2,861,613 | 430.9 |
| H011 | 8,327 | 16,254 | 6,188,290 | 380.7 |
| H012 | 2,289 | 7,203 | 2,386,452 | 331.3 |
| H013 | 3,025 | 6,118 | 2,185,135 | 357.2 |
| H014 | 2,221 | 5,790 | 2,237,670 | 386.5 |
| H015 | 3,976 | 7,009 | 2,944,857 | 420.2 |
| H016 | 8,152 | 20,493 | 8,147,320 | 397.6 |
| H017 | 1,565 | 3,244 | 1,042,278 | 321.3 |
| H018 | 6,303 | 14,768 | 6,169,277 | 417.7 |
| H019 | 10,001 | 17,719 | 7,165,649 | 404.4 |
| H020 | 3,052 | 5,145 | 2,096,401 | 407.5 |
| Mean | 4,561.7 | 8,605.5 | 3,505,898 | 407.4 |
| SD | 2,326.4 | 4,693.4 | 1,870,983 | 35.4 |
| Total | 91,234 | 172,109 | 7,011,7961 |  |

PBMCs, peripheral blood mononuclear cells; SD, standard deviation

| **Table S3. Numbers of unique reads, reads and nucleotides in TRB reads obtained from PBMCs of 20 healthy individuals** | | | | |
| --- | --- | --- | --- | --- |
| **Healthy individual** | **Total number of unique reads** | **Total number of reads** | **Total number of nucleotides** | **Mean number of nucleotides per read** |
| H001 | 3,092 | 4,007 | 1,626,917 | 406.0 |
| H002 | 2,069 | 3,624 | 1,620,164 | 447.1 |
| H003 | 979 | 3,602 | 1,595,988 | 443.1 |
| H004 | 3,025 | 3,664 | 1,637,322 | 446.9 |
| H005 | 1,275 | 1,970 | 884,000 | 448.7 |
| H006 | 1,274 | 2,122 | 952,553 | 448.9 |
| H007 | 3,301 | 3,760 | 1,665,584 | 443.0 |
| H008 | 2,089 | 3,956 | 1,737,410 | 439.2 |
| H009 | 2,664 | 3,575 | 1,595,609 | 446.3 |
| H010 | 2,761 | 3,384 | 1,517,514 | 448.4 |
| H011 | 5,198 | 8,182 | 3,369,499 | 411.8 |
| H012 | 4,882 | 11,759 | 4,421,616 | 376.0 |
| H013 | 4,272 | 8,117 | 2,793,454 | 344.1 |
| H014 | 2,119 | 4,652 | 1,578,364 | 339.3 |
| H015 | 3,735 | 5,298 | 1,929,452 | 364.2 |
| H016 | 2,663 | 4,086 | 1,582,494 | 387.3 |
| H017 | 2,348 | 4,341 | 1,676,546 | 386.2 |
| H018 | 3,044 | 4,807 | 1,804,197 | 375.3 |
| H019 | 4,317 | 5,923 | 2,285,404 | 385.9 |
| H020 | 2,875 | 4,099 | 1,639,809 | 400.1 |
| mean | 2,899.1 | 4,746.4 | 1,895,695 | 409.4 |
| SD | 1,156.2 | 2,280.0 | 806,581 | 37.8 |
| Total | 57,982 | 94,928 | 37,913,896 |  |

PBMCs, peripheral blood mononuclear cells; SD, standard deviation.

| **Table S4. Percentage of mismatched nucleotides in in-frame and out-of-frame TCR sequences** | | | | | | | | | | | | | | | | | | | | | |
| --- | --- | --- | --- | --- | --- | --- | --- | --- | --- | --- | --- | --- | --- | --- | --- | --- | --- | --- | --- | --- | --- |
| **Healthy individuals** | **% Mismatched nucleotides** | | | | | | | | | | | | | | | | | | | | |
|  | **TRAV** | | |  | | **TRAJ** | | |  | | | **TRBV** | | |  | | | **TRBJ** | | |  |
|  | **all** | **in-frame** | **out-of-frame** |  | **all** | | **in-frame** | **out-of-frame** | |  | **all** | | **in-frame** | **out-of-frame** | |  | **all** | | **in-frame** | **out-of-frame** |  |
| H001 | 0.54 | 0.51 | 0.67 |  | 0.40 | | 0.32 | 0.78 | |  | 0.58 | | 0.54 | 0.71 | |  | 0.54 | | 0.35 | 1.16 |  |
| H002 | 0.92 | 0.90 | 0.99 |  | 0.61 | | 0.39 | 1.25 | |  | 0.89 | | 0.83 | 1.17 | |  | 0.36 | | 0.24 | 0.96 |  |
| H003 | 0.93 | 0.89 | 1.04 |  | 0.63 | | 0.41 | 1.28 | |  | 0.83 | | 0.77 | 1.14 | |  | 0.36 | | 0.23 | 0.98 |  |
| H004 | 0.93 | 0.88 | 1.07 |  | 0.60 | | 0.40 | 1.22 | |  | 0.85 | | 0.79 | 1.14 | |  | 0.40 | | 0.24 | 1.12 |  |
| H005 | 0.87 | 0.81 | 1.05 |  | 0.61 | | 0.39 | 1.22 | |  | 0.90 | | 0.84 | 1.22 | |  | 0.37 | | 0.26 | 0.95 |  |
| H006 | 0.89 | 0.86 | 0.98 |  | 0.56 | | 0.36 | 1.19 | |  | 0.77 | | 0.71 | 1.08 | |  | 0.34 | | 0.22 | 1.13 |  |
| H007 | 0.89 | 0.83 | 1.07 |  | 0.59 | | 0.36 | 1.22 | |  | 0.80 | | 0.72 | 1.19 | |  | 0.46 | | 0.30 | 1.19 |  |
| H008 | 0.89 | 0.85 | 1.02 |  | 0.61 | | 0.41 | 1.31 | |  | 0.89 | | 0.83 | 1.14 | |  | 0.49 | | 0.28 | 1.30 |  |
| H009 | 0.93 | 0.87 | 1.11 |  | 0.63 | | 0.40 | 1.29 | |  | 0.91 | | 0.84 | 1.24 | |  | 0.39 | | 0.26 | 1.04 |  |
| H010 | 0.88 | 0.85 | 0.97 |  | 0.61 | | 0.37 | 1.26 | |  | 0.85 | | 0.80 | 1.16 | |  | 0.35 | | 0.21 | 1.05 |  |
| H011 | 0.54 | 0.50 | 0.63 |  | 0.48 | | 0.35 | 0.85 | |  | 0.54 | | 0.51 | 0.66 | |  | 0.52 | | 0.36 | 1.08 |  |
| H012 | 0.58 | 0.50 | 0.83 |  | 0.65 | | 0.46 | 1.22 | |  | 0.61 | | 0.56 | 0.75 | |  | 0.69 | | 0.48 | 1.32 |  |
| H013 | 0.58 | 0.51 | 0.79 |  | 0.56 | | 0.36 | 1.18 | |  | 0.57 | | 0.52 | 0.70 | |  | 0.64 | | 0.42 | 1.27 |  |
| H014 | 0.68 | 0.62 | 0.90 |  | 0.48 | | 0.34 | 1.00 | |  | 0.54 | | 0.50 | 0.68 | |  | 0.59 | | 0.40 | 1.19 |  |
| H015 | 0.65 | 0.61 | 0.84 |  | 0.40 | | 0.31 | 0.89 | |  | 0.57 | | 0.53 | 0.68 | |  | 0.70 | | 0.46 | 1.33 |  |
| H016 | 0.55 | 0.49 | 0.72 |  | 0.50 | | 0.35 | 0.94 | |  | 0.57 | | 0.52 | 0.74 | |  | 0.59 | | 0.38 | 1.23 |  |
| H017 | 0.57 | 0.53 | 0.69 |  | 0.53 | | 0.38 | 0.94 | |  | 0.57 | | 0.53 | 0.70 | |  | 0.57 | | 0.38 | 1.27 |  |
| H018 | 0.51 | 0.46 | 0.68 |  | 0.47 | | 0.38 | 0.81 | |  | 0.56 | | 0.52 | 0.70 | |  | 0.53 | | 0.34 | 1.18 |  |
| H019 | 0.53 | 0.45 | 0.79 |  | 0.44 | | 0.32 | 0.86 | |  | 0.63 | | 0.58 | 0.76 | |  | 0.66 | | 0.41 | 1.37 |  |
| H020 | 0.47 | 0.41 | 0.74 |  | 0.43 | | 0.31 | 0.96 | |  | 0.54 | | 0.50 | 0.66 | |  | 0.50 | | 0.31 | 1.15 |  |
| mean | 0.72 | 0.67 | 0.88 |  | 0.54 | | 0.37 | 1.08 | |  | 0.70 | | 0.65 | 0.91 | |  | 0.50 | | 0.33 | 1.16 |  |
| SD | 0.18 | 0.19 | 0.16 |  | 0.08 | | 0.04 | 0.19 | |  | 0.15 | | 0.14 | 0.24 | |  | 0.12 | | 0.08 | 0.13 |  |

SD, standard deviation.

| **Table S5. Occurrence frequency of out-of-frame unique sequence reads in TRA and TRB** | | |
| --- | --- | --- |
| **Healthy individual** | **%Frequency** | |
|  | **TRA** | **TRB** |
| H001 | 21.9 | 27.0 |
| H002 | 35.8 | 26.1 |
| H003 | 52.3 | 42.7 |
| H004 | 27.8 | 20.7 |
| H005 | 33.8 | 22.3 |
| H006 | 31.8 | 19.8 |
| H007 | 28.6 | 19.5 |
| H008 | 31.0 | 27.4 |
| H009 | 31.2 | 21.5 |
| H010 | 30.6 | 19.1 |
| H011 | 29.1 | 27.5 |
| H012 | 41.5 | 46.0 |
| H013 | 33.4 | 38.1 |
| H014 | 29.4 | 39.4 |
| H015 | 21.1 | 34.3 |
| H016 | 34.3 | 32.1 |
| H017 | 32.7 | 33.2 |
| H018 | 27.9 | 29.2 |
| H019 | 27.4 | 32.1 |
| H020 | 21.7 | 28.3 |
| mean | 31.2 | 29.3 |
| SD | 7.0 | 7.9 |

SD, standard deviation.

| **Table S6. Percentage frequency of shared TRA reads between all pairs of individuals** | | | | | | | | | | | | | | | | | | | | |
| --- | --- | --- | --- | --- | --- | --- | --- | --- | --- | --- | --- | --- | --- | --- | --- | --- | --- | --- | --- | --- |
|  | **H001** | **H002** | **H003** | **H004** | **H005** | **H006** | **H007** | **H008** | **H009** | **H010** | **H011** | **H012** | **H013** | **H014** | **H015** | **H016** | **H017** | **H018** | **H019** | **H020** |
| **H001** | – | 0.71 | 0.29 | 1.04 | 1.29 | 0.80 | 1.61 | 0.78 | 0.89 | 0.84 | 1.12 | 0.87 | 0.73 | 0.77 | 1.28 | 0.92 | 0.77 | 1.05 | 1.13 | 1.11 |
| **H002** | 0.34 | – | 0.47 | 0.59 | 0.37 | 0.37 | 0.51 | 0.27 | 0.72 | 0.36 | 0.23 | 0.31 | 0.26 | 0.23 | 0.25 | 0.33 | 0.32 | 0.35 | 0.26 | 0.46 |
| **H003** | 0.08 | 0.28 | – | 0.21 | 0.15 | 0.23 | 0.04 | 0.21 | 0.16 | 0.12 | 0.08 | 0.13 | 0.10 | 0.00 | 0.15 | 0.07 | 0.19 | 0.10 | 0.10 | 0.13 |
| **H004** | 0.98 | 1.17 | 0.70 | – | 1.05 | 1.05 | 1.32 | 1.25 | 1.41 | 1.24 | 0.82 | 1.00 | 0.79 | 0.68 | 1.26 | 0.72 | 0.83 | 0.78 | 0.78 | 0.79 |
| **H005** | 0.71 | 0.43 | 0.29 | 0.61 | – | 0.89 | 0.90 | 0.84 | 0.79 | 0.74 | 0.59 | 0.66 | 0.50 | 0.45 | 0.80 | 0.58 | 0.89 | 0.71 | 0.53 | 0.69 |
| **H006** | 0.58 | 0.57 | 0.59 | 0.81 | 1.17 | – | 1.01 | 0.63 | 1.07 | 0.92 | 0.82 | 1.05 | 0.63 | 0.54 | 0.93 | 0.75 | 0.89 | 0.75 | 0.57 | 0.75 |
| **H007** | 1.49 | 1.00 | 0.12 | 1.29 | 1.51 | 1.29 | – | 1.13 | 1.31 | 1.28 | 0.94 | 1.00 | 0.69 | 1.04 | 1.58 | 0.88 | 1.28 | 1.14 | 1.05 | 1.05 |
| **H008** | 0.44 | 0.32 | 0.41 | 0.75 | 0.86 | 0.49 | 0.70 | – | 0.58 | 0.66 | 0.36 | 0.48 | 0.26 | 0.36 | 0.68 | 0.47 | 0.32 | 0.41 | 0.38 | 0.43 |
| **H009** | 0.75 | 1.28 | 0.47 | 1.25 | 1.20 | 1.24 | 1.19 | 0.87 | – | 1.18 | 0.77 | 0.87 | 0.63 | 0.63 | 1.18 | 0.80 | 0.89 | 0.81 | 0.60 | 0.92 |
| **H010** | 0.71 | 0.64 | 0.35 | 1.11 | 1.14 | 1.08 | 1.17 | 0.99 | 1.19 | – | 0.90 | 0.96 | 0.79 | 0.59 | 1.16 | 0.74 | 0.64 | 0.95 | 0.86 | 0.85 |
| **H011** | 1.58 | 0.68 | 0.41 | 1.22 | 1.51 | 1.59 | 1.43 | 0.90 | 1.29 | 1.49 | – | 1.66 | 6.02 | 0.99 | 2.04 | 1.37 | 1.21 | 1.52 | 1.49 | 1.38 |
| **H012** | 0.34 | 0.25 | 0.18 | 0.41 | 0.46 | 0.56 | 0.42 | 0.33 | 0.40 | 0.44 | 0.46 | – | 0.36 | 0.09 | 0.58 | 0.44 | 0.58 | 0.48 | 0.42 | 0.36 |
| **H013** | 0.37 | 0.28 | 0.18 | 0.43 | 0.46 | 0.45 | 0.38 | 0.24 | 0.38 | 0.48 | 2.19 | 0.48 | – | 0.41 | 0.58 | 0.33 | 0.38 | 0.38 | 0.46 | 0.56 |
| **H014** | 0.29 | 0.18 | 0.00 | 0.27 | 0.31 | 0.28 | 0.42 | 0.24 | 0.28 | 0.26 | 0.26 | 0.09 | 0.30 | – | 0.50 | 0.33 | 0.26 | 0.17 | 0.35 | 0.29 |
| **H015** | 0.86 | 0.36 | 0.35 | 0.90 | 0.98 | 0.87 | 1.15 | 0.81 | 0.95 | 0.92 | 0.97 | 1.00 | 0.76 | 0.90 | – | 0.82 | 0.89 | 0.84 | 0.95 | 0.85 |
| **H016** | 1.27 | 0.96 | 0.35 | 1.06 | 1.45 | 1.43 | 1.32 | 1.13 | 1.31 | 1.20 | 1.35 | 1.57 | 0.89 | 1.22 | 1.69 | – | 0.83 | 1.19 | 1.20 | 0.66 |
| **H017** | 0.20 | 0.18 | 0.18 | 0.23 | 0.43 | 0.33 | 0.37 | 0.15 | 0.28 | 0.20 | 0.23 | 0.39 | 0.20 | 0.18 | 0.35 | 0.16 | – | 0.17 | 0.20 | 0.26 |
| **H018** | 1.12 | 0.78 | 0.35 | 0.88 | 1.38 | 1.10 | 1.32 | 0.78 | 1.03 | 1.20 | 1.15 | 1.31 | 0.79 | 0.50 | 1.33 | 0.92 | 0.70 | – | 1.18 | 0.92 |
| **H019** | 1.91 | 0.93 | 0.59 | 1.40 | 1.63 | 1.34 | 1.92 | 1.13 | 1.21 | 1.71 | 1.79 | 1.83 | 1.52 | 1.58 | 2.39 | 1.47 | 1.28 | 1.87 | – | 1.77 |
| **H020** | 0.58 | 0.50 | 0.23 | 0.43 | 0.65 | 0.54 | 0.59 | 0.39 | 0.56 | 0.52 | 0.50 | 0.48 | 0.56 | 0.41 | 0.65 | 0.25 | 0.51 | 0.44 | 0.54 | – |

| **Table S7. Percentage frequency of shared TRB reads between all pairs of individuals** | | | | | | | | | | | | | | | | | | | | |
| --- | --- | --- | --- | --- | --- | --- | --- | --- | --- | --- | --- | --- | --- | --- | --- | --- | --- | --- | --- | --- |
|  | **H001** | **H002** | **H003** | **H004** | **H005** | **H006** | **H007** | **H008** | **H009** | **H010** | **H011** | **H012** | **H013** | **H014** | **H015** | **H016** | **H017** | **H018** | **H019** | **H020** |
| **H001** | – | 0.05 | 0.00 | 0.00 | 0.08 | 0.00 | 0.03 | 0.05 | 0.11 | 0.07 | 0.08 | 0.02 | 0.05 | 0.00 | 0.00 | 0.11 | 0.00 | 0.00 | 0.12 | 0.07 |
| **H002** | 0.03 | – | 0.00 | 0.03 | 0.08 | 0.00 | 0.00 | 0.05 | 0.04 | 0.00 | 0.02 | 0.02 | 0.00 | 0.00 | 0.00 | 0.04 | 0.04 | 0.00 | 0.02 | 0.00 |
| **H003** | 0.00 | 0.00 | – | 0.00 | 0.00 | 0.00 | 0.00 | 0.00 | 0.00 | 0.00 | 0.00 | 0.00 | 0.00 | 0.00 | 0.00 | 0.00 | 0.00 | 0.00 | 0.02 | 0.00 |
| **H004** | 0.00 | 0.05 | 0.00 | – | 0.16 | 0.00 | 0.03 | 0.05 | 0.08 | 0.07 | 0.00 | 0.02 | 0.00 | 0.05 | 0.00 | 0.08 | 0.04 | 0.03 | 0.02 | 0.00 |
| **H005** | 0.03 | 0.05 | 0.00 | 0.07 | – | 0.00 | 0.00 | 0.00 | 0.04 | 0.00 | 0.00 | 0.00 | 0.00 | 0.00 | 0.03 | 0.04 | 0.04 | 0.00 | 0.00 | 0.03 |
| **H006** | 0.00 | 0.00 | 0.00 | 0.00 | 0.00 | – | 0.03 | 0.00 | 0.04 | 0.04 | 0.02 | 0.02 | 0.00 | 0.05 | 0.11 | 0.00 | 0.04 | 0.00 | 0.02 | 0.00 |
| **H007** | 0.03 | 0.00 | 0.00 | 0.03 | 0.00 | 0.08 | – | 0.05 | 0.04 | 0.04 | 0.02 | 0.02 | 0.00 | 0.00 | 0.00 | 0.04 | 0.00 | 0.00 | 0.00 | 0.03 |
| **H008** | 0.03 | 0.05 | 0.00 | 0.03 | 0.00 | 0.00 | 0.03 | – | 0.00 | 0.04 | 0.02 | 0.00 | 0.00 | 0.00 | 0.03 | 0.11 | 0.00 | 0.00 | 0.02 | 0.00 |
| **H009** | 0.10 | 0.05 | 0.00 | 0.07 | 0.08 | 0.08 | 0.03 | 0.00 | – | 0.11 | 0.06 | 0.02 | 0.00 | 0.00 | 0.03 | 0.04 | 0.00 | 0.03 | 0.02 | 0.00 |
| **H010** | 0.06 | 0.00 | 0.00 | 0.07 | 0.00 | 0.08 | 0.03 | 0.05 | 0.11 | – | 0.00 | 0.02 | 0.00 | 0.09 | 0.03 | 0.11 | 0.00 | 0.00 | 0.07 | 0.07 |
| **H011** | 0.13 | 0.05 | 0.00 | 0.00 | 0.00 | 0.08 | 0.03 | 0.05 | 0.11 | 0.00 | – | 0.04 | 0.16 | 0.00 | 0.08 | 0.11 | 0.00 | 0.16 | 0.05 | 0.14 |
| **H012** | 0.03 | 0.05 | 0.00 | 0.03 | 0.00 | 0.08 | 0.03 | 0.00 | 0.04 | 0.04 | 0.04 | – | 0.30 | 0.14 | 0.21 | 0.45 | 0.26 | 0.03 | 0.02 | 0.00 |
| **H013** | 0.06 | 0.00 | 0.00 | 0.00 | 0.00 | 0.00 | 0.00 | 0.00 | 0.00 | 0.00 | 0.13 | 0.27 | – | 0.14 | 0.16 | 0.19 | 0.13 | 0.03 | 0.02 | 0.03 |
| **H014** | 0.00 | 0.00 | 0.00 | 0.03 | 0.00 | 0.08 | 0.00 | 0.00 | 0.00 | 0.07 | 0.00 | 0.06 | 0.07 | – | 0.00 | 0.04 | 0.21 | 0.00 | 0.00 | 0.03 |
| **H015** | 0.00 | 0.00 | 0.00 | 0.00 | 0.08 | 0.31 | 0.00 | 0.05 | 0.04 | 0.04 | 0.06 | 0.16 | 0.14 | 0.00 | – | 0.15 | 0.09 | 0.03 | 0.02 | 0.00 |
| **H016** | 0.10 | 0.05 | 0.00 | 0.07 | 0.08 | 0.00 | 0.03 | 0.14 | 0.04 | 0.11 | 0.06 | 0.25 | 0.12 | 0.05 | 0.11 | – | 0.13 | 0.03 | 0.02 | 0.03 |
| **H017** | 0.00 | 0.05 | 0.00 | 0.03 | 0.08 | 0.08 | 0.00 | 0.00 | 0.00 | 0.00 | 0.00 | 0.12 | 0.07 | 0.24 | 0.05 | 0.11 | – | 0.00 | 0.00 | 0.00 |
| **H018** | 0.00 | 0.00 | 0.00 | 0.03 | 0.00 | 0.00 | 0.00 | 0.00 | 0.04 | 0.00 | 0.10 | 0.02 | 0.02 | 0.00 | 0.03 | 0.04 | 0.00 | – | 0.14 | 0.10 |
| **H019** | 0.16 | 0.05 | 0.10 | 0.03 | 0.00 | 0.08 | 0.00 | 0.05 | 0.04 | 0.11 | 0.04 | 0.02 | 0.02 | 0.00 | 0.03 | 0.04 | 0.00 | 0.20 | – | 0.10 |
| **H020** | 0.06 | 0.00 | 0.00 | 0.00 | 0.08 | 0.00 | 0.03 | 0.00 | 0.00 | 0.07 | 0.08 | 0.00 | 0.02 | 0.05 | 0.00 | 0.04 | 0.00 | 0.10 | 0.07 | – |

**Figure S1.**


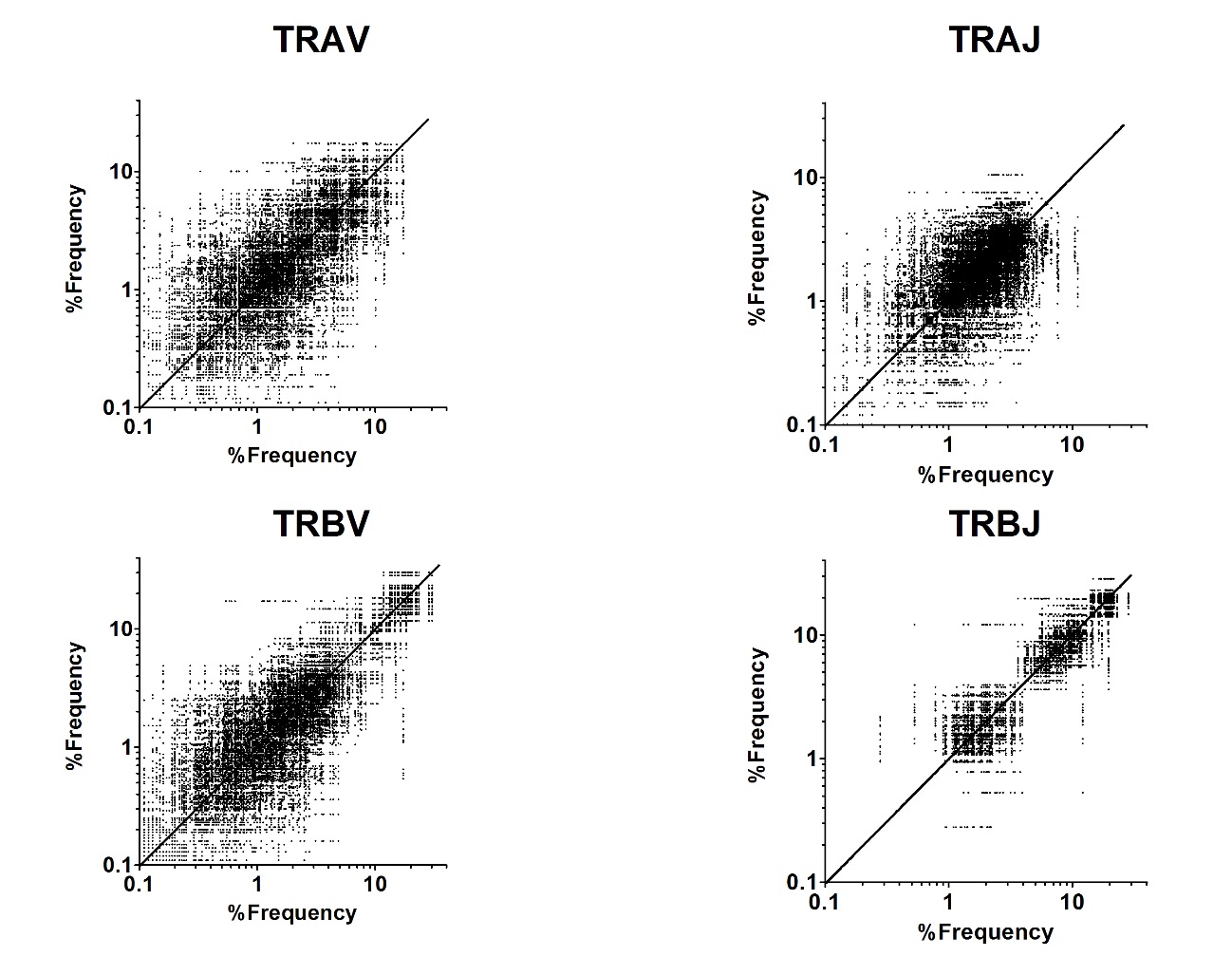


**Correlation of gene usage of TRAV, TRAJ, TRBV and TRBJ between healthy individuals.**

Percentage frequencies of TRAV (upper left), TRAJ (upper right), TRBV (lower left) and TRBJ (lower right) between all pairs of individuals are plotted. A dot with lower deviation from diagonal line (y=x) indicates a better correlation.

**Figure S2.**

**Concordance correlation coefficient in TRAV, TRAJ, TRBV and TRBJ.**

The correlation coefficient between two samples from healthy individuals was calculated by Spearman’s correlation test. Each dot indicates a correlation coefficient value between a pair of individuals. Mean correlation coefficient is shown as a red horizontal bar (n=190)

**Figure S3**

**
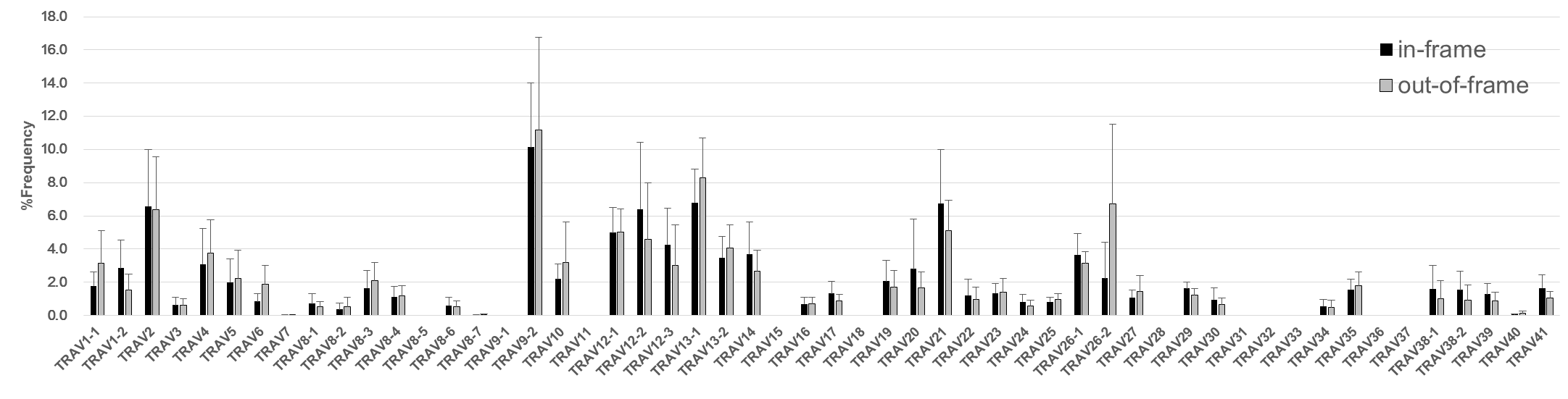
A**

**B**


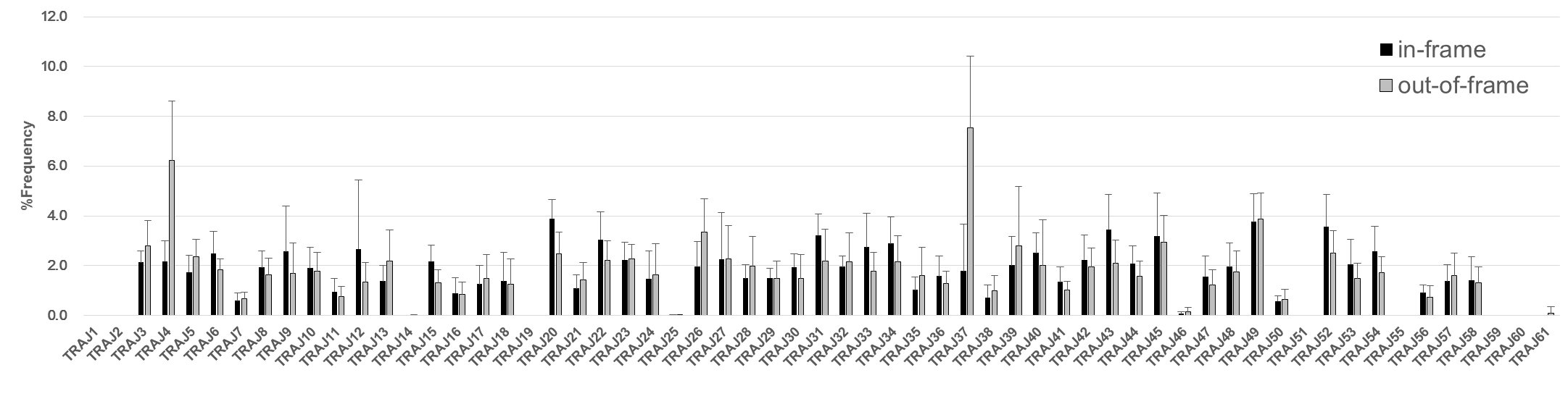


**Figure S3, continued**

**C**


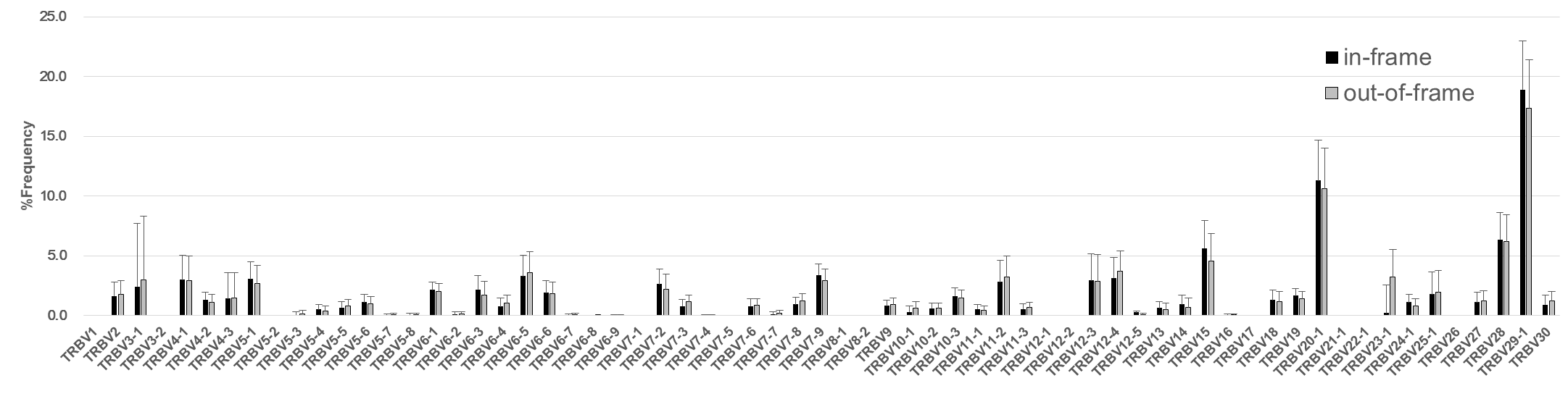


**Comparison of TCR usages between in-frame and out-of-frame reads sequences**

Mean percentage frequencies and standard deviations (error bars) of TRAV (A), TRAJ (B), TRBV (C) and TRBJ (D) in in-frame reads (black) and out-of-frame (grey) were shown (n = 20). On the whole, similar usages of TCR were observed between in-frame and out-of-frame reads. On the other hand, there were differences in the frequencies of TRAV26-2, TRAJ4, TRAJ37, TRBV23-1, TRBJ1-4 and TRBJ2-2 between in-frame and out-of-frame reads.

**D**


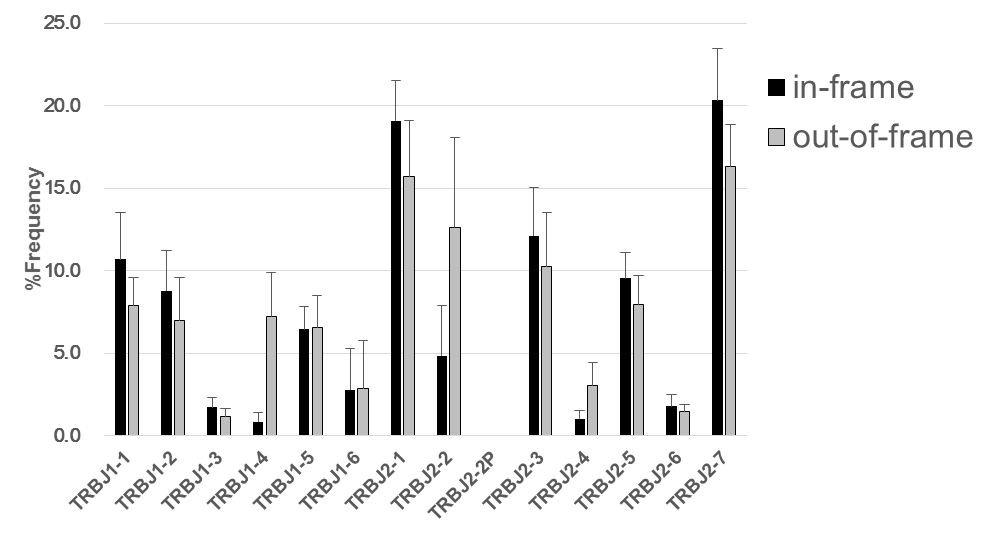


**Figure S4**

**Diversity indices of in-frame and out-of-frame TRA and TRB.**

Inverse Simpson index (A) and Shannon-Weaver Index (B) were compared between in-frame (IN) and out-of-frame (OUT) reads of TRA and TRB. The Shannon diversity indices were significantly higher in in-frame reads than in out-of-frame reads (TRA: 7.37 ± 0.72 vs. 6.81 ± 0.49, p < 0.05, TRB: 7.05 ± 0.64 vs. 6.46 ± 0.60, P < 0.005). However, there was no difference in the inverse Simpson index between in-frame and out-of-frame reads.

**Figure S5**

**Correlation of TCR diversity with age.**

Diversity indices (A: Shannon index in TRA, B: Shannon index in TRB, C: Inverse Simpson index in TRA, D: Inverse Simpson index in TRB) were plotted against individual’s age (n=20). Each dot indicates each healthy individual. There was a significant correlation of Shannon index in TRA with age (P<0.05, Spearman’s correlation) but no correlation of the other indices with age. Overall, there were tendencies for inverse relationship of diversity indices with age.

**Figure S6**

**Correlation of TCR usage from a published FACS data with AL-PCR and Multiplex PCR**

Mean percentage frequencies of each TRBV with FACS were plotted against that with AL-PCR (n=20) (A) or that with Multiplex PCR (n=3) (B). Correlation coefficients and p values were shown in figures. For datasets with flow cytometric analysis, data published by van der Beemd et al. (van den Beemd, Cytometry, 40:336-345, 2000) were used. Datasets were obtained with PBMCs from 3 Japanese healthy individuals with a multiplex PCR method by Adaptive Biotechnologies Inc. (Seattle, WA) in this study. There was a better correlation of FACS data with AL-PCR than Multiplex-PCR
